# Carbon emissions from clinical activities by speciality in secondary and tertiary care in England: an exploratory cross-sectional analysis of routine administrative data

**DOI:** 10.1016/j.lanepe.2025.101333

**Published:** 2025-06-02

**Authors:** Hasina Begum, William K. Gray, Robin M. Simpson, Rose Ingleton, Manraj K. Phull

**Affiliations:** a Greener NHS, NHS England, London, UK; b Getting It Right First Time Programme, NHS England, London, UK; c Barking, Havering and Redbridge University Hospitals NHS Trust, London, UK

**Keywords:** Environmental sustainability, Carbon footprint, Sustainable healthcare, Planetary health

## Abstract

**Background:**

The National Health Service (NHS) in England has committed to achieving net zero carbon emissions by 2045. A key early step in this journey is to understand where opportunities to decarbonise healthcare exist. The aim of this paper is to explore the potential to use available activity and emissions intensity data to investigate the carbon emissions of different specialty-level clinical activities in secondary and tertiary care in the NHS in England.

**Methods:**

This was an exploratory, cross-sectional analysis of routine administrative data from secondary and tertiary care in the NHS in England. We included data for all patients admitted to hospital (including outpatient attendances, but excluding emergency attendances without subsequent admission) in England during the financial year 2022/23. The Hospital Episodes Statistics dataset and Theatre Productivity Data Collection were used. Carbon emissions factors were taken from published sources and linked to activity volumes to quantify the carbon emissions at a clinical activity level.

**Findings:**

Data for 17,024,278 hospital admissions and 101,973,593 outpatient attendances were analysed. Outpatient attendances accounted for 45% of the measured carbon emissions. Of the remaining 55% relating to admitted patient care, emergency admissions accounted for 45% (82% of admitted patient care), in-patient elective activity 7% and day case activity 3%. The top 20 clinical specialties accounted for 79% of the carbon emissions, with general internal medicine, trauma and orthopaedics and general surgery the three highest carbon emitting specialties.

**Interpretation:**

These data provide insight into the carbon emissions of specific elements of secondary and tertiary care activity in England. Such activity-level (and even more granular procedure-level and patient pathway-level) analysis is needed to inform carbon hotspot identification, intervention development and implementation to reduce the carbon emissions of care. As more granular data become available (e.g., on pharmaceutical use), such estimates will become more comprehensive.

**Funding:**

This research received no specific grant from any funding agency in the public, commercial, or not-for-profit sectors.

**Research in context**

**Evidence before this study**

Organisational or system level carbon emissions estimation has almost exclusively been done through modelling expenditure data. Activity based methods have rarely been employed but offer the possibility of more granular estimates that provide insight into carbon hotspots and therefore can inform mitigation strategies. We searched PubMed on 30th September, 2024 for articles that reported the carbon footprint of health services using clinical activity data. We used the search terms (“carbon footprint” or “carbon emissions”) and (“clinical activity”) in the abstract or title. This returned two studies, only one of which was relevant. This was a Spanish study of the carbon emissions of a single hospital. At 46% of total emissions, electricity was the largest component. The study did not consider emissions for individual specialties.

**Added value of this study**

We evaluated the carbon footprint of components of secondary and tertiary healthcare specialties in England using clinical activity data. The measured carbon footprint was composed of: outpatient activity (45%), emergency admissions (45%), elective in-patient admissions (7%) and elective day case admissions (3%). The top 20 clinical specialties accounted for 79% of the carbon emissions, with general internal medicine, trauma and orthopaedics and general surgery the three highest carbon emitting specialties.

**Implications of all the available evidence**

Clinical activity can be used to understand the contribution of different elements of healthcare to the overall carbon footprint. This is an important first step in developing interventions to reduce the carbon footprint of health services.

## Introduction

Climate change poses a formidable threat to global health and the ability of health systems to deliver essential services in both the near- and long-term.^1^ Healthcare itself has a larger carbon footprint than aviation and shipping combined, responsible for an estimated 4.4–5.2% of global carbon emissions.^2^, ^3^, ^4^, ^5^ Additionally, climate change mitigation policies and actions stand to bring health co-benefits by leveraging the health payoffs from improved air quality, healthier diets, and a more physically active population.^6^ Consequently, in October 2020 the National Health Service (NHS) in the United Kingdom (UK) became the first national health system in the world to commit to decarbonise its operations. The NHS in England set a clear target for net zero by 2045 for its total carbon footprint, with an 80% reduction by 2036–39.^7^ A commitment that gained legislative footing in 2022 through the Health and Care Act.^8^

The mitigation strategies supporting tangible action in key areas such as the supply chain, estates and facilities, travel and transport and emissions from medicines^9^ have been shaped by national-level data on emissions and the ability to monitor progress.^10^ The Greener NHS programme is coordinating this work in England. The NHS has reduce its emissions by 30% since 2010, although much of this improvement has come from decarbonization of UK energy supply rather than through healthcare focused decarbonisation.^11^

To build on and maximise these gains the NHS must engage with frontline clinicians by applying a clinical lens to carbon emission assessments and reduction strategies. Investigating the emissions profile of different clinical specialties can help achieve this while also ensuring alignment with wider health service priorities (e.g., equity of access, optimal patient outcomes, service efficiency) and supporting necessary changes in clinical decision making.

Hospital-based secondary and tertiary care services are estimated to account for about half of the NHS’ carbon emissions.^10^ Referral into secondary and tertiary care is often for specialist review, planned treatment or emergency care. Delivery of this care is usually disaggregated by clinical specialty, echoing the way health care professionals are trained and specialise and how clinical pathways, departments and wards are set up and run within the NHS.

This paper aims to investigate the carbon emissions of secondary and tertiary care clinical activities at a specialty level within the NHS in England. Investigating the carbon emissions of a health system in this way provides new understanding and allows identification of carbon hotspots and actions to reduce carbon emissions whilst ensuring the delivery of high-quality equitable care.

## Methods

### Study design

This was an exploratory cross-sectional analysis using administrative data.

### Ethics

The presentation of data follows current NHS England guidance for use of Hospital Episodes Statistics (HES) data for research purposes.^12^ Consent from individuals involved in this study was not required for analysis of this administrative dataset. Ethical approval was not sought for the present study because it did not directly involve human participants. This study was completed in accordance with the Helsinki Declaration as revised in 2013.

### Setting and timing

Data were extracted for the whole of England for the period 1st April 2022 to 31st March 2023 (inclusive). This period was chosen as the most recent full financial year of data available at the time of extraction.

### Data sources, data extraction and exclusions

The primary data source was the Hospital Episode Statistic (HES) database for England; mandated data collection for all NHS secondary and tertiary activity in England. HES includes patient-level data for all patients admitted to hospital (admitted patient care) and outpatient attendances, including unplanned/emergency admissions. HES data are entered by trained clinical coders in every NHS hospital trust in England and are collated by NHS England. Data from HES were used to estimate the volume and type of in-hospital care delivered to patients, extracted at the clinical specialty-level. The list of specialty titles and codes has been developed, and is maintained, by the General and Specialty Medical Practice.^13^

Office of Populations Censuses and Surveys Classification of Interventions and Procedures version 4 (OPCS-4) codes were used to identify the type of procedures and interventions carried out during a patient's admission or outpatient attendance. The OPCS-4 codes were truncated to three characters, placing the procedures into broad categories. For elective hospital admissions, the first three OPCS-4 codes and for emergency (non-elective) admissions the first ten OPCS-4 codes were extracted and used to define the activity undertaken during a hospital spell (the period from admission to discharge) for each patient. For elective procedures, the dominant procedure code will generally appear within the first three positions. For emergency care this is often not the case, given the unplanned nature of the care and so an extended number of codes were included. Thus, the vast majority of relevant procedures/interventions will have been captured. Data were excluded for non-NHS patients (not recorded in HES) and NHS-funded patients treated in non-NHS hospitals (non-NHS setting care). Emergency department attendances with no subsequent hospital admission (not recorded in HES), and non-attended outpatient attendances (not recorded in HES) were not counted. Although non-admitted emergency department attendances are not recorded in HES, patients admitted treated and discharged on the same day (same-day emergency care) are recorded. Days were counted as calendar days, exact time of admission was not recorded.

To estimate time in theatre for each included surgical procedure, data were extracted from NHS England's Theatre Productivity Data Collection (TPDC). The TPDC provides details on surgical procedures conducted in England that require an operating theatre. The TPDC is not mandatory. During the study period, coverage of TPDC was around 80% of all theatre-based procedures undertaken in England. It includes details of the NHS hospital trust, hospital site, surgical team, procedure undertaken and timing of key events (e.g., anaesthesia induction, procedure start, procedure finish, into recovery room) during the procedure. The length of procedure was calculated as the difference in time from when a patient entered the anaesthetic room to entering the recovery room. Procedures were identified from the primary three-character OPCS-4 procedure code. As the data were non-normally distributed, the median length of procedure was used.

### Activity categories

Clinical specialties were defined according to those used by NHS England within the HES dataset and detailed in its data dictionary.^13^ The activity identified through the OPCS-4 codes in HES was categorised as described below:

For hospital admissions, all OPCS-4 codes identified in the procedural record for each episode of admitted patient care recorded in HES were reviewed and categorised as:

1. Surgical procedures: These were subdivided according to the three categories described by Abbott et al:^14^
  a. ‘Restrictive’ category including major procedures that due to duration or complexity may often result in tissue injury.
  b. ‘Intermediate’ category including procedures routinely undertaken in an operating theatre and/or under general or regional anaesthesia. Procedures already defined as ‘restrictive’ were excluded from this category.
  c. ‘Inclusive’ category comprising procedures that might be considered surgery, including minor surgery, interventional radiology procedures and diagnostic endoscopies, but excluding non-invasive diagnostic procedures (e.g., diagnostic imaging). Procedures already defined as ‘restrictive’ or ‘intermediate’ were excluded from this category.
2. Non-surgery intervention or treatment: This category comprised interventions or treatments not considered as surgery according to any of Abbott et al.‘s definitions. This includes care delivered on the ward, in specialist units and those requiring specialist input such as renal dialysis, radiotherapy, chemotherapy, electroconvulsive therapy (often performed in the anaesthetic room), labour and delivery and blood transfusion.
3. Diagnostic test: Including diagnostic imaging and tests not covered by any previous category.

Subsidiary or qualifying OPCS-4 codes, which are designed to be used in conjunction with main procedure codes to provide more detail or context (e.g., body site, laterality), were excluded from any analysis given their supplementary nature. A full break down of all three-character OPCS-4 codes into these categories is given in Supplementary Materials Tables S1–S7.

For outpatient data, OPCS-4 codes were not useful as a means of refining outpatient activity recorded in HES due to issues of data quality; thus these data were not subdivided. In 2022/23, 68.8% of outpatient attendances had no OPCS-4 codes recorded.^15^ Outpatient care and activities typically consist of appointments for clinical consultation, minor surgical procedures, diagnostic, imaging and blood tests, pre- and post-operative assessments, post-discharge checks, routine assessments for chronic conditions and allied healthcare professional, nurse and midwife led assessment and treatment. Both face-to-face and virtual (e.g., telephone, online) outpatient appointments are captured in HES.

### Carbon emissions factors

Carbon emissions factors were applied to building block elements of each patient's care to calculate carbon equivalent emissions (CO_2_e) by multiplying an activity volume by the relevant carbon emissions factor. Carbon emissions factors have been taken from various published sources and internal NHS England calculations. No cradle-to-grave new life cycle assessments (LCAs) were conducted as part of the study. Where available, ISO 14067 or 14040 compliant factors have been used. Details of the source of each carbon emissions factor are given in Supplementary Material Table S8. Each factor comes with its own methodological approach to estimation and limitations. All carbon emissions factors were validated for use by members of NHS England's Greener NHS team. The most up-to-date and robust carbon emissions factors available were used. Some carbon emissions factors are based on studies in single organisations or were derived some time ago. Some are taken from non-UK settings, but were checked for relevance and validity and adapted where possible. Proxies taken from similar clinical activities were used where specific carbon emissions factors were not available. In some cases, activity OPCS-4 codes were excluded from the analysis due to a lack of available carbon emissions factors.

The carbon emissions factors were mapped to each activity element described below and then multiplied by the volume of activity to give the CO_2_e for that activity. The various activity elements were then aggregated as required (e.g., by clinical specialty). Brief details on the carbon emissions factors used are presented below and in Table 1 and further details given in Supplementary Material Table S8.

**Table 1:** Procedure categorisation and carbon mapping process for interventions and treatments.

Category	Sub-category activity description for carbon emissions factor allocation	Activity	Carbon emission factor (kgCO_2_e)
Surgical procedure: Restrictive, in theatre	A. Major surgery under general anaesthesia	Surgical procedure	Anaesthetic gases: 17 kgCO_2_e^a^ Energy use per minute of procedure: 0.197 kgCO_2_e^16^ Consumables: 7.1 kgCO_2_e Equipment: 1.3 kgCO_2_e Water and waste: 0.2 kgCO_2_e (Average factor used if length of procedure not available: 38.4 kgCO_2_e^17^)
Surgical procedure: Intermediate, in theatre	A. Surgery under general anaesthesia	Surgical procedure	Anaesthetic gases: 17 kgCO_2_e^a^ Energy use per minute of procedure: 0.197 kgCO_2_e^16^ Consumables: 7.1 kgCO_2_e Equipment: 1.3 kgCO_2_e Water and waste: 0.2 kgCO_2_e (Average factor used if length of procedure not available: 38.4 kgCO_2_e^17^)
	B. Surgery under local or regional anaesthesia	Surgical procedure	Energy use per minute of procedure: 0.197 kgCO_2_e^b^^,^^16^ Consumables: 7.1 kgCO_2_e Equipment: 1.3 kgCO_2_e Water and waste: 0.2 kgCO_2_e (Average factor used if length of procedure not available: 21.4 kgCO_2_e)
Surgical procedure: Inclusive, outside of theatre	A. Procedure performed in a specialist suite/unit/procedure room.	Endoscopy	15.4 kgCO_2_e^18^
		Interventional radiology	Energy use per minute of procedure: 0.197 kgCO_2_e^b^^,^^16^ Equipment: 1.3 kgCO_2_e Water and waste: 0.2 kgCO_2_e (Average factor used if length of procedure not available: 21.4 kgCO_2_e)
		Minor procedure performed in procedure room.	Energy use per minute of procedure: 0.197 kgCO_2_e^b^^,^^16^ Consumables: 7.1 kgCO_2_e Equipment: 1.3 kgCO_2_e Water and waste: 0.2 kgCO_2_e (Average factor used if length of procedure not available: 21.4 kgCO_2_e)
	B. Procedure performed outside of the operating theatre in ward or clinic setting.	Minor procedure performed in ward or clinic setting.	Energy use per minute of procedure: 0.033 kgCO_2_e^c^^,^^16^ Consumables: 7.1 kgCO_2_e Equipment (1.3 kgCO_2_e) Water and waste (0.2 kgCO_2_e) (Average factor used if length of procedure not available: 11.6 kgCO_2_e)
Non-surgical intervention or treatment, outside of theatre	A. Procedure, treatment, or intervention performed on a specialist unit and or requiring specialist input	Dialysis	14.8 kgCO_2_e^19^
		Radiotherapy	30.6 kgCO_2_e^20^
		Electroconvulsive therapy	Anaesthetic gases: 17 kgCO_2_e^a^ Energy use per minute of procedure: 0.197 kgCO_2_e^d^^,^^16^ (Average factor used if length of procedure not available: 31.1 kgCO_2_e)
		Childbirth (delivery)	17 kgCO_2_e^21^
	B. Procedure, treatment, or intervention that can be delivered on the ward and is deemed part of ward-based care	Blood transfusion	7.56 kgCO_2_e^22^
		Pharmacological treatment	Excluded as no appropriate carbon emission factor or carbon accounting approach available at time of study.
		Other ward care	Captured in in-patient bed day carbon emission factor.
Diagnostic test	A. Diagnostic imaging and testing	Radiological imaging and investigations	9.2 kgCO_2_e^23^
		Blood tests	0.3 kgCO_2_e^24^
		Other diagnostic tests	Excluded as no appropriate carbon emission factor or carbon accounting approach available at time of study.

a Desflurane was excluded given very limited use within the NHS during the study period.

b Energy use is deemed to be equivalent to that of an operating room given the specialist/imaging equipment used and has been adjusted by length of procedure.

c Energy use reduced by a sixth to account for out of theatre energy utilisation.

d Electroconvulsive therapy is mostly performed in the anaesthetic room and is not a surgical procedure therefore consumables, equipment and water and waste associated with surgical procedures have been excluded.

For admitted patient care, the following elements were considered.

1. Activity type: A carbon emissions factor was applied to the activity undertaken during the hospital spell based on the categories outlined above. The construction of the carbon emissions factor for each activity type is shown in Table 1.
2. Length of stay: For the period from admission to discharge a carbon emissions factor of 37.9 kgCO_2_e was applied to each day's stay in a general and acute bed.^25^ A carbon emissions factor of 89.5 kgCO_2_e was applied to each day's stay in a critical care (intensive care unit (ICU) or high dependence unit (HDU)) bed.^25^
3. Travel: For elective admission a travel carbon emissions factor was calculated by multiplying the average travel distance for the clinical specialty the patient was admitted under by 0.25 kgCO_2_e/mile and then multiplying by two for a round trip. The emissions intensity per mile was calculated using UK National Travel Survey^26^ data for travel modes used for healthcare purposes and Department for Energy Security and Net Zero emissions factors.^27^ The average travel distance in miles for each specialty was calculated using the UK Department for Transport reference dataset methodology based on the mean travel distance during 2022/23 from the patient's Lower Super Output Area (LSOA) of residence to the hospital site to which they were admitted.^28^ Where travel distance data for a clinical specialty could not be obtained, the average distance across all specialties (9.2 miles) was assigned. For emergency admissions, arrival by ambulance was assumed and a travel carbon emissions factor of 0.8 kgCO_2_e/mile (calculated based on ambulance fuel consumption data) applied to the inbound journey and 0.25 kgCO_2_e/mile applied to the return journey. An average distance to hospital of 8.8 miles was applied to both journeys based on administrative data.
4. Emergency department visit: Where a patient was admitted into hospital as an emergency they were assigned an emergency department attendance carbon emissions factor of 13.8 kgCO_2_e.^29^

For outpatient attendances, the carbon emissions estimate was derived from a single building block element; whether the attendances was face-to-face (carbon emissions factor: 22.0 kgCO_2_e) or virtual (carbon emissions factor: 0.1 kgCO_2_e).^30^

### Statistical methods and data analysis

Data were extracted using SQL Server Management Studio (v19) and analysed using Microsoft Excel (both Microsoft Corp, Redmond, WA, USA). Data analysis is entirely descriptive and standard summary statistics are used. Data are primarily categorised by main clinical specialty for presentation.

The degree of uncertainty around the value of the various carbon emissions factors is likely to be by far the biggest source of uncertainty in our carbon emissions estimates. Most of the carbon emissions factors used do not include a robust measure of uncertainty. This makes evaluation of the uncertainty inherent in our estimates complex, as noted by other authors.^10^ To overcome this issue, we have use a one-way (or one-at-a-time) sensitivity analysis and systematically varied the two most influential carbon emissions estimates (hospital bed days and out-patient attendances) by ±20% and detailed the impact of this on specialty level totals. This method is recommended by Hamby et al.^31^

### Role of the funding source

This research received no specific grant from any funding agency in the public, commercial, or not-for-profit sectors. The corresponding author had full access to all the data in the study and had final responsibility for the decision to submit for publication.

## Results

Data were available for 17,024,278 episodes of admitted patient care and 101,973,593 outpatient attendances during the 12-month study period. Table 2 and Supplementary Material Figure S1 (admitted patient care) and Table 3 and Supplementary Material Figure S2 (outpatient attendances) present a breakdown of activity volumes by setting and specialty. The majority of admissions were either day-case or emergency (48% and 46% respectively). Face-to-face attendances made up 79% of outpatient attendances. General internal medicine had by far the largest number of admitted patients and allied health professionals the largest number of outpatient attendances. The top 20 specialties by admissions and outpatient attendances represent 81% of all activity for both groups.

**Table 2:** Admitted patient care volume by top 20 specialties, 2022/23.

Specialty	Elective	Day case	Emergency/non-elective	Unknown admission method	Total activity volume	Total carbon emissions (kilotonnes CO_2_e)
General internal medicine	30,057	443,142	1,780,968	342	2,254,509	504
General surgery	181,675	798,379	701,615	212	1,681,881	189
Paediatrics	32,166	145,146	1,160,177	4041	1,341,530	156
Gastroenterology	26,327	1,082,312	84,172	96	1,192,907	52
Renal Medicine	14,319	803,029	70,542	41	887,931	42
Trauma & orthopaedics	202,881	269,671	288,831	24	761,407	156
Obstetrics	33,793	36,036	688,672	842	759,343	70
Clinical haematology	28,536	690,152	35,044	652	754,384	26
Gynaecology	83,804	150,870	441,580	174	676,428	54
Medical oncology	14,879	572,492	38,975	50	626,396	14
Clinical oncology	14,129	563,313	38,598	5	616,045	15
Urology	109,107	348,954	146,190	51	604,302	40
Ophthalmology	12,096	512,715	16,199	2	541,012	14
Emergency medicine	860	2156	442,487	6	445,509	42
Geriatric medicine	4726	15,747	398,588	23	419,084	174
Cardiology	38,412	186,113	157,512	24	382,061	54
Acute internal medicine	2496	24,723	326,299	32	353,550	74
Respiratory medicine	19,979	81,668	190,261	20	291,928	69
Ear nose and throat	47,982	129,004	87,685	7	264,678	21
Midwifery	2079	578	259,771	339	262,767	21
All other specialties	194,658	1,249,380	459,350	3238	1,906,626	447
Total	**1,094,961**	**8,105,580**	**7,813,516**	**10,221**	**17,024,278**	**2180**

**Table 3:** Outpatient attendances volume by top 20 specialties, 2,022,122.

Specialty	Face-to-face attendance	Virtual attendance	Total activity volume	Total carbon emissions (kilotonnes CO_2_e)
Allied health professional	9,033,988	1,600,704	10,634,692	199
Trauma & orthopaedics	6,161,944	1,003,487	7,165,431	136
Ophthalmology	6,184,067	450,375	6,634,442	136
Nursing	4,516,871	1,959,433	6,476,304	99
General surgery	4,457,290	1,350,348	5,807,638	98
Midwifery	3,980,419	206,190	4,186,609	88
General internal medicine	2,647,812	1,176,207	3,824,019	58
Gynaecology	3,039,547	591,370	3,630,917	67
Paediatrics	3,058,857	547,636	3,606,493	67
Cardiology	2,591,911	1,009,046	3,600,957	57
Obstetrics	2,741,883	312,105	3,053,988	60
Dermatology	2,741,009	310,912	3,051,921	60
Urology	1,953,181	1,077,710	3,030,891	43
Clinical haematology	1,885,526	1,093,029	2,978,555	42
Ear nose and throat	2,324,900	395,512	2,720,412	51
Gastroenterology	1,290,712	1,309,156	2,599,868	28
Rheumatology	1,762,184	699,266	2,461,450	39
Respiratory medicine	1,561,979	746,967	2,308,946	34
Clinical oncology	1,516,701	788,891	2,305,592	33
Neurology	1,427,135	636,082	2,063,217	31
All other specialties	15,909,927	3,921,324	19,831,251	349
**Total**	**80,787,843**	**21,185,750**	**101,973,593**	**1777**

For admitted patient care, the carbon emissions of the top 20 specialties by volume is shown in Table 2; 15 of the top 20 specialties by activity volume were also in the top 20 for carbon emissions. The five specialties in the top 20 for carbon emissions that did not appear in the top 20 for activity volume were adult mental illness, endocrinology and diabetes, forensic psychiatry, neurosurgery, and cardiothoracic surgery. The relatively high carbon emissions of geriatric medicine relative to activity volume reflects relatively long hospital stays in this specialty.

The carbon emissions of the top 20 outpatient specialties by activity volume is shown in Table 3. The carbon emissions largely mirrored the activity volume data, with only minor changes depending on the proportion of activity that was virtual. For outpatient activity, the top 20 specialties by volume of activity were the same as the top 20 specialties for carbon emissions, other than gastroenterology (21st place on carbon emissions) being replaced by radiology (carbon emissions: 35 kilotonnes of CO_2_e).

**Fig. 1:**
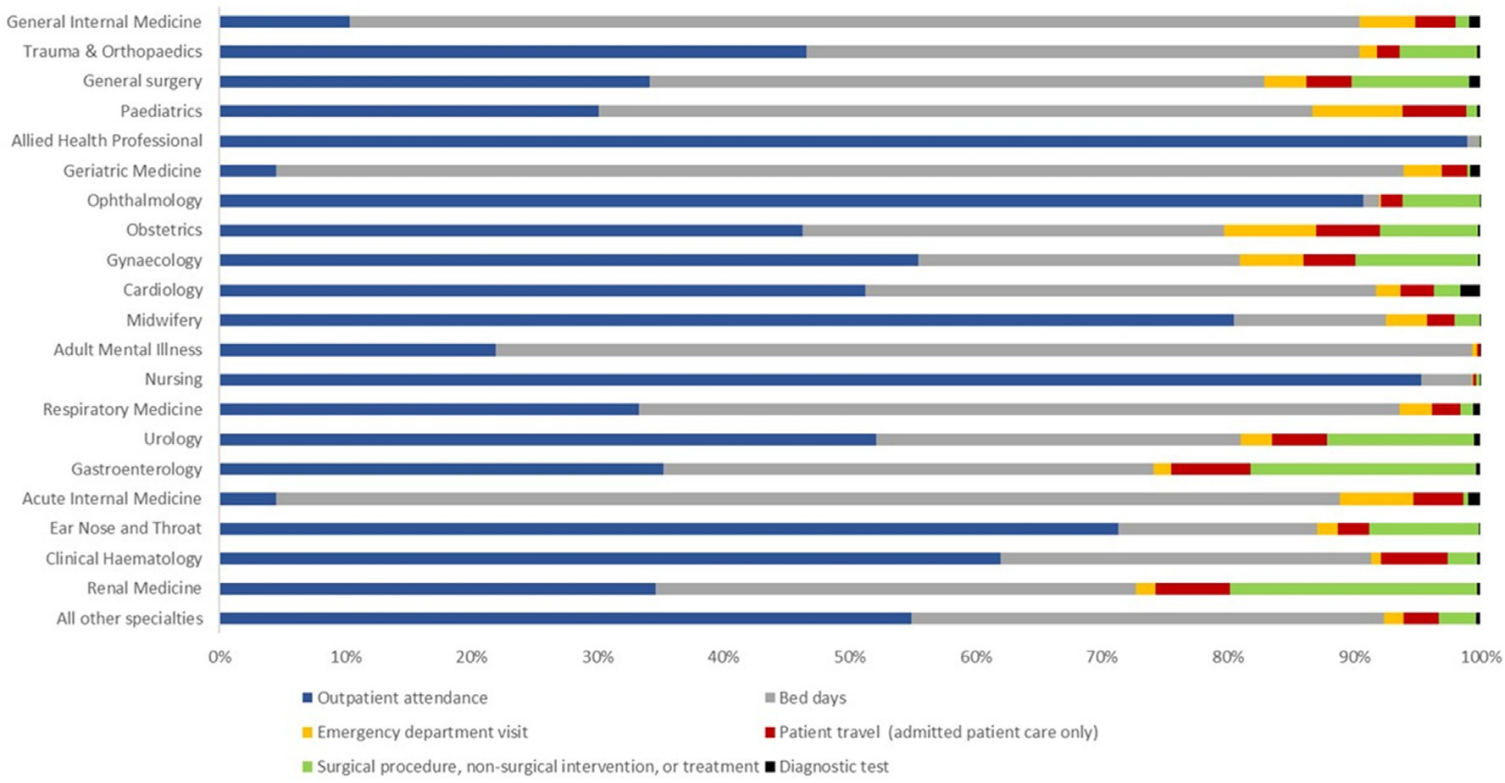
**Top 20 specialties by carbon emissions for financial year 2022/23, hospital admissions and outpatient attendances combined**.

The combined carbon emissions for admitted patient care and outpatient attendances, with breakdown for specific elements of care for the top 20 specialties by carbon emissions, is shown in Fig. 1 and the total carbon emissions for the top 20 specialties by admission/attendance method and activity are shown in Table 4 and Supplementary Material Figure S3. Data on the carbon emissions of surgery, interventions, treatment and diagnostic tests are further broken down in Table 5. Outpatient patient travel related carbon emissions could not be disaggregated from the carbon emissions factor used and therefore is captured within the total for outpatient care.

**Table 4:** Carbon emissions of secondary and tertiary care in England by category.

	Total carbon emissions (kilotonnes CO_2_e)
**Activity type**	
Outpatient attendances	1777 (45%)
Bed days	1780 (45%)
Emergency department visits^a^	108 (3%)
Patient travel (admitted patient care only)	115 (3%)
Surgical procedure, non-surgical intervention, or treatment	160 (4%)
Diagnostic test	18 (0.5%)
**Admission/attendance method**	
Emergency	1781 (45%)
Elective	259 (7%)
Day case	130 (3%)
Outpatients	1777 (45%)
Unknown admission method	11 (0.3%)
**Specialty**	
General internal medicine	562 (14%)
Trauma & orthopaedics	291 (7%)
General surgery	287 (7%)
Paediatrics	223 (6%)
Allied health professional	201 (5%)
Geriatric medicine	183 (5%)
Ophthalmology	150 (4%)
Obstetrics	130 (3%)
Gynaecology	121 (3%)
Cardiology	111 (3%)
Midwifery	109 (3%)
Adult mental illness	105 (3%)
Nursing	104 (3%)
Respiratory medicine	103 (3%)
Urology	83 (2%)
Gastroenterology	81 (2%)
Acute internal medicine	78 (2%)
Ear nose and throat	72 (2%)
Clinical haematology	67 (2%)
Renal medicine	63 (2%)
All other specialties	833 (21%)

a Emergency department visits are only counted where this results in an admission to hospital.

**Table 5:** Breakdown of the carbon emissions of surgical procedure, non-surgical intervention and treatment and diagnostic tests.

	Carbon emissions (kilotonnes CO_2_e)		
	Elective and day-case	Emergency	Total
**Surgery with anaesthetic gas**	54.1	24.1	78.2
**Surgery without anaesthetic gas**	14.9	2.7	17.6
**Procedures performed outside of theatre**			
Endoscopy	28.7	1.6	30.3
Minor procedure with anaesthetic gases (e.g., Electroconvulsive therapy in anaesthetic room)	2.8	6.8	9.5
Minor procedure without anaesthetic gases	1.5	0.2	1.7
Interventional radiology	0.2	0.2	0.5
**Non-surgical therapeutic procedure or treatment**			
Blood transfusion	1.4	0.2	1.6
Dialysis	12.8	0.7	13.5
Childbirth	0.1	4.4	4.5
Radiotherapy	2.4	0.1	2.5
**Diagnostic test**			
Blood test	0.1	<0.1	0.1
Imaging	2.4	15.5	17.9

**Fig. 2:**
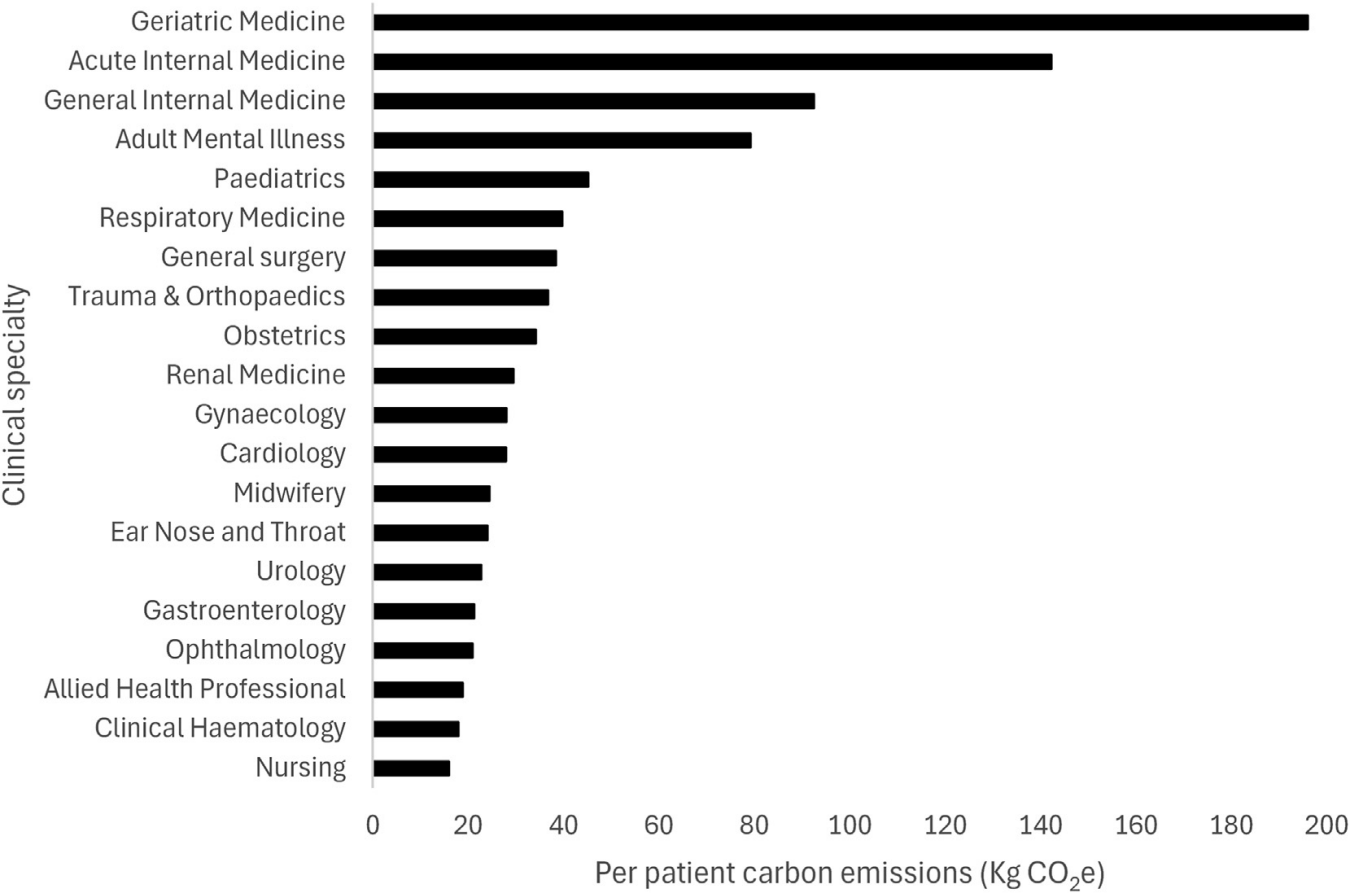
**Per patient carbon emissions of top 20 specialties by total carbon emissions for financial year 2022/23, hospital admissions and outpatient attendances combined**.

The top 20 specialties accounted for 79% of carbon emissions across all specialties, with general internal medicine, trauma and orthopaedics and general surgery the three specialties with the highest emissions. When considering the per-patient carbon footprint for these specialties (Fig. 2), geriatric medicine, acute internal medicine and general internal medicine had the largest emissions. A full breakdown of total carbon emissions for each specialty and each type of activity and per-patient is given in Supplementary Material Table S9.

The results of the one-way sensitivity analyses are presented in Supplementary Material Table S10. Varying the bed day and outpatient attendance carbon emissions factors systematically had a very limited impact on the composition of the clinical specialties in the top 20 and on their relative order.

## Discussion

This paper explores the potential to use administrative data on clinical activity to estimate the carbon emissions associated with different activities by specialty within secondary and tertiary care provision in England. These estimates were calculated by combining patient care data, from a clinically relevant and widely available administrative database, with carbon emissions factors taken from published literature and Greener NHS data modelling.

The methodology was designed specifically such that secondary and tertiary care emissions were allocated at a clinical specialty and activity level rather than to hospital workstreams and systems. As a result, we were able to identify which specialties had the most significant carbon impact and highlight the respective contributions of different clinical activities and interventions. Categorising carbon emissions by clinical specialty ensures that they align with the categorisations of activity used within hospitals in England and are meaningful. Although our findings in isolation do not lead to obvious decarbonisation actions, extending this work to look at individual pathways and procedures within a specialty can provide this insight. Some of this work has already been started by NHS England's Getting It Right First Time programme within the urology specialty and a guide to decarbonising the bladder cancer management pathway has been published.^32^, ^33^, ^34^, ^35^ Equipping the clinical community with this understanding should ensure that decarbonisation strategies are considered and delivered alongside the core priorities of delivering high-quality, safe, efficient, cost effective and equitable care, whilst maintaining and or improving patient outcomes and experience. There is growing evidence demonstrating that this is feasible and that decarbonisation actions are likely to directly align and support these wider strategies and healthcare aims.^34^^,^^36^, ^37^, ^38^

Granular carbon footprinting at a procedure or patient pathway level will be needed to identify more specific carbon hotspots and interventions to further reduce carbon emissions, implement low carbon solutions and monitor progress. Our approach and findings serve as a starting point and proof of concept of how such footprinting can be performed. Building on this study, individual procedures within each specialty should be explored and more detailed (e.g., using LCA assessment where needed) emissions modelling undertaken.

Specialties with the highest carbon emissions were often those with the largest activity volumes (e.g. general internal medicine, allied healthcare professionals). However, some specialties had higher carbon emissions than might be expected from their activity volumes (e.g., adult mental illness, geriatric medicine, neurosurgery) and this is seen in the per-patient emissions data. In these cases, contributing factors included disproportionate high resource-utilisation or treating a patient population frequently requiring long inpatient stays. Indeed, the specialties with the highest per-patient carbon footprint were generally those with high average in-patient bed days per patient.

Secondary and tertiary care specialties share many common activities, practices, and processes within their patient care pathways. By understanding the carbon impact of these common themes, proposed sustainability initiatives can be adapted for cross-specialty implementation. Additionally, we highlight some key sub-specialty activities demonstrating a significant carbon footprint which warrant additional focus and targeted action.

Outpatient activity was a key contributor to the carbon emissions for secondary and tertiary care, accounting for almost half of the total carbon emissions. The two clinical specialties with the highest volume of outpatient activity were ophthalmology and trauma and orthopaedics. Allied healthcare professionals and nursing specialties also had high outpatient activity, likely representative of these professionals working across different specialties. However, the entirety of the involvement of these professional groups across all clinical activity will not be captured within data recorded in HES. As examples, nursing support in theatres, on wards and in critical care and allied healthcare professional involvement in ward-based rehabilitation will be captured within each specialty rather than separately.

The granularity of outpatient data was limited, detailing only activity volumes, clinical specialty and whether they were in-person or by virtual consultation. We are unable to report these data broken down by activity type (e.g., consultation, diagnostics/imaging, treatment). Greater granularity in these data are needed and would be invaluable in understanding the carbon emissions of outpatient activity and identifying carbon hotspots. It should be noted that whilst outpatient activity has a significant carbon contribution this is largely driven by volume of activity and that per unit of activity it is of low carbon intensity.

Admitted patient care accounted for 55% of the total estimated carbon emissions. This represents an essential area to target for decarbonisation with the potential for major impact across almost all clinical specialties. A key driver of carbon emissions in this category was in-patient bed days. Reducing unnecessary bed days can be enabled by adhering to best practice guidance and pathways and supporting early safe discharge.^7^^,^^39^ Beyond this, further research into the key components of in-patient stay that lead to high carbon emissions is needed.

Emergency/non-elective routes accounted for 46% of the activity volume, but 82% of the carbon emissions of admitted patient care. These emissions may be amenable to reduction through initiatives such as optimising the entry route to secondary/tertiary care provision, a shift towards preventative care models, comprehensive long-term condition management, early recognition of patient deterioration necessitating an escalation in care and patient empowerment to support better self-management.^40^ There will also need to be improved joining up of care between primary and secondary healthcare providers, ensuring the right care is delivered in the right place at the right time.^41^

Critical care represents a particularly difficult area to target given that unnecessary admission or prolonged length of stay may seem less amenable to change in this setting. Nevertheless, there are initiatives such as Critical Care Outreach services, enhanced recovery pathways and prehabilitation for surgical patients and timely step-down or use of high acuity wards which support the prevention of unnecessary admissions, readmissions and lengthy stays.^42^ The implementation of a low-carbon critical care environment is possible. Five key components to address this have already been identified by professionals within this specialty area including: drugs, consumables, energy and water, waste and avoidance of futile treatment.^42^^,^^43^

Surgical procedures and interventions accounted for 4% of the overall secondary and tertiary care carbon emissions and 7% of the admitted patient care emissions. The Green Surgery Report^37^ and the Intercollegiate Green Theatre Checklist^44^ are examples of evidence-based, peer-reviewed resources outlining actionable steps and recommendations to decarbonise surgical care. Key hotspots identified include anaesthesia, single-use products, and energy consumption.

Endoscopies had one of the largest carbon emissions of any activity sub-group within admitted patient care. Although performed out of theatre, these are resource-intensive procedures performed by many specialties. The combination of high activity volume, multi-specialty and high resource use make endoscopy an ideal area to target for decarbonisation.^45^ This same set of attributes is likely to apply to interventional radiology, a procedure-based specialty demonstrating increasing patient demand year-on-year.^46^ The gastroenterology clinical community have led the way towards green endoscopy by mapping out the practical measures endoscopy units can take to improve their environmental sustainability.^47^

Dialysis also had relatively large carbon emissions. The renal community have set clear net zero targets at a specialty level and are implementing initiatives to minimise their environmental impact such as the development of an in–centre haemodialysis carbon calculator, a kidney unit sustainability champions scheme and supply chain decarbonisation actions.^48^

Patient travel is a common activity across all specialties and settings. Although the data suggests that travel is a relatively small source of emissions, we were only able to disaggregate it for admitted patient care, as travel was included in the outpatient carbon emissions factor used. Since outpatient activity represents 86% of all activity recorded and travel will be a disproportionately large proportion of the carbon emissions of outpatient care, the true carbon emissions of travel is likely to be a much larger proportion of the overall secondary and tertiary care emissions than reported here. Furthermore, visitor travel was not included in our estimate.

As well as decarbonising modes of transportation,^49^ strategies to decarbonise travel should include minimising the need to travel. Initiatives such as one-stop pre-assessment, patient initiated follow up and virtual appointments should be used where feasible.^37^ Specialties where this is likely to be a key hotspot and area of focus are tertiary departments with centralised patient services, necessitating long travel distances for many patients.

The extent of data coverage is the most important limitation of our study. We investigated the main sources of carbon emissions for different clinical specialties and activity categories. It is not an attempt to calculate the total carbon emissions associated with secondary and tertiary healthcare in the NHS in England.

Previous research has estimated that pharmaceuticals (medicines, chemicals, inhalers and anaesthetic gases) account for approximately 25% of the total carbon footprint of the NHS in England.^7^ Due to a lack of specific carbon emissions factor data in the literature and detailed recording of prescribing and pharmaceutical drug use within HES,^50^ we were unable to account for this in our study. This clearly represents a major data exclusion and limitation of this study. This topic requires renewed focus by the relevant stakeholders for meaningful interpretation and action.

Only a small proportion of the carbon emissions associated with pathology and imaging is likely to be included in our analysis.^25^ The ability to account for any additional testing on an individual basis is limited to only those recorded using the OPCS-4 classification and this is reflected in the relatively small contribution of diagnostics tests to our estimates.

The annual number of radiological investigations in England has been reported as 40 million, with some evidence of unnecessary imaging.^51^ Imaging can have a significant environmental impact with some routine modalities such as magnetic resonance imaging having a carbon footprint as high as 17.5 kgCO_2_e per scan, largely due to high energy requirements.^23^ Similarly, the carbon emissions of routine pathology services (e.g., general phlebotomy, histopathology) could not be calculated accurately as relatively few of these investigations are coded as procedures within the HES dataset. Reports suggest that approximately 800 million laboratory tests are performed each year within the NHS with up to 40% of these thought to be clinically unnecessary.^52^ This area offers the potential for significant carbon savings given the volume of potentially avoidable tests currently being performed. The associated financial savings will likely be substantial. Addressing this issue of excessive, unnecessary testing requires the implementation of standardised, evidence-based clinical pathways to ensure that investigations are requested only when clinically indicated.^53^, ^54^, ^55^ Where investigations are required, their application should align with low-carbon principles, focusing on clinical efficiency, resource-utilisation, waste management and energy usage.^23^

We only included data for emergency department attendances if they resulted in admission to hospital. This will have resulted in an underestimate of the carbon emissions associated with emergency care. Future work should look to build emergency department attendance data into such modelling.

The issues of data coverage detailed above are likely to mean that some clinical specialties and care categories have lower reported carbon emissions than their true values. In general terms, emissions estimates for elective surgical care will be less influenced by the lack of medicines and emergency care data than specialties more focused on medical and emergency care. Respiratory medicine (e.g., inhaler use, emergency attendance) and geriatric medicine (e.g., polypharmacy, emergency attendance) are two specialties where impacts are likely to be largest.

Some additional limitations are also acknowledged. While the most up-to-date and robust carbon emissions factors available, and validated for use by Greener NHS, have been used, some of these are based on studies in single organisations or were derived some time ago. In addition, proxies are used for activity where specific carbon emissions factors are not available (see Supplementary Material Table S8 for more details) and, in some cases, activity OPCS-4 codes were excluded from the analysis due to a lack of published available carbon emissions factors (e.g., pharmacological treatment and some specific diagnostic tests, see Supplementary Material Tables S5 and S6 for more details). Similarly, in the absence of suitable published carbon emissions factors, internal Greener NHS estimates were used; we recognize that these have not undergone external peer review. As more up-to-date and granular carbon intensity figures become available, they can be incorporated into future analyses. We also acknowledge that our analysis is necessarily an over-simplification, given the generic nature of the carbon emissions factors used and the aggregation of activity data. This generic approach is necessary given the breadth of the data used but will have resulted in some biases. As an example, the use of generic estimates for patient travel will underestimate emissions for rural dwellers. More detail is needed when examining individual care pathways and procedures; this more detailed approach has been used in the case of bladder cancer pathways and will be rolled out through the GIRFT programme to many other pathways.^35^

We note previous reports of data quality issues within HES,^56^ although these are unlikely to result in significant bias in our findings, which rely on broad classifications of specialty and procedures. Although detailed information on data quality within the TPDC are not currently available, similar limitations of data quality apply.

The vast majority of carbon emissions factors had limited information on the uncertainty of the estimates, and this made assessing the uncertainty within our estimates difficult. This issue has also been highlighted by Tennison et al.^10^ Future work on individual procedures should look to build on what we have done and further investigate key sensitivities and uncertainties around specific estimates. Such work has already been undertaken by our team in relation to bladder cancer pathways.^35^

### Conclusion

This methodology demonstrates the key carbon hotspots and drivers for carbon emissions within care pathways at a clinical activity and specialty level within secondary and tertiary level care in England. It highlights the positive correlation between volume of activity and carbon emissions whilst also identifying specific carbon intensive activities that warrant targeted action. We acknowledge the need to expand the methodology to allow a more detailed carbon analysis at an individual patient pathway level, improve data collection on outpatient activity and access to pharmacy and diagnostic (pathology and imaging) datasets as well as the creation of a standardised approach to calculating clinical activity carbon footprints. This will allow a more complete and more granular picture to be developed.

## Contributors

This study was designed and organised by HB, WKG, RS, RI and MP. Data cleaning and analysis was by HB and WKG. Writing of the first draft was by MP, HB, RI and WKG. All authors critically reviewed the manuscript and agreed to submission of the final draft.

## Data sharing statement

WKG and HB and RS had full access to all the data in the study and take responsibility for the integrity of the data and the accuracy of the data analysis. This report does not contain patient identifiable data. Data in this report are anonymised. The underlying HES data cannot be made available directly by the authors as the data were obtained under licence/data sharing agreement from NHS England. HES data are available from NHS England upon application.

## Declaration of interests

The authors declare that there is no conflict of interest.
