# Supplementary material for: Carbon emissions from clinical activities by speciality in secondary and tertiary care in England: an exploratory cross-sectional analysis of routine administrative data
Source: Lancet Reg Health Eur. 2025 Jun 2;54:101333. doi: 10.1016/j.lanepe.2025.101333 (PMC12167066; doi:10.1016/j.lanepe.2025.101333)

**Supplementary material**

**Table of Contents**

[**Supplementary material Table S1: OPCS-4.10 codes included in the restrictive category for surgery.** 2](#_Toc196307547)

[**Supplementary material Table S2: OPCS-4.10 codes included in the intermediate A category (Surgery under general anaesthesia)** 3](#_Toc196307548)

[**Supplementary material Table S3: OPCS-4.10 codes included in the intermediate B category (Surgery under local or regional anaesthesia)** 4](#_Toc196307549)

[**Supplementary material Table S4: OPCS-4.10 codes included in the inclusive A and B categories (Procedure performed outside of theatre in a specialist suite, unit, procedure room, ward or other clinic setting.)** 5](#_Toc196307550)

[**Supplementary material Table S5: OPCS-4.10 codes included in the Non-surgical intervention or treatment, outside of theatre A and B categories (Procedure, treatment, or intervention performed on a specialist unit and or requiring specialist input and procedure, treatment, or intervention that can be delivered on the ward and is deemed part of ward-based care)** 6](#_Toc196307551)

[**Supplementary material Table S6: OPCS-4.10 codes included in diagnostic test category.** 7](#_Toc196307552)

[**Supplementary material Table S7: OPCS-4.10 codes excluded as deemed subsidiary / qualifying.** 8](#_Toc196307553)

[**Supplementary material Table S8: Carbon factors applied to each activity element and information source** 9](#_Toc196307554)

[**Supplementary material Table S9: Carbon footprint (kilotonnes CO_2_e) breakdown by specialty and activity type** 14](#_Toc196307555)

[**Supplementary material Table S10: Sensitivity analyses for specialty level totals** 17](#_Toc196307556)

[**Supplementary material Figure S1: Admitted patient care volume by top 20 specialties, 2022/23.** 18](#_Toc196307557)

[**Supplementary material Figure S2: Outpatient attendances volume by top 20 specialties, 2022/23.** 19](#_Toc196307558)

[**Supplementary material Figure S3: Carbon emissions of secondary and tertiary care in England by category.** 20](#_Toc196307559)

# **Supplementary material Table S1: OPCS-4.10 codes included in the restrictive category for surgery.**

All codes below were assigned a surgical carbon emission factor which included anaesthetic gas emissions, energy use adjusted for length of procedure (determined using the Theatre Productivity Data Collection, where available), consumables, equipment, water, and waste.

| A01 | B16 | G02 | G82 | J31 | K36 | L38 | M61 | S55 | V18 | W21 | X08 |
| --- | --- | --- | --- | --- | --- | --- | --- | --- | --- | --- | --- |
| A02 | B18 | G03 | H01 | J32 | K37 | L41 | M62 | T01 | V19 | W22 | X09 |
| A03 | B20 | G04 | H02 | J33 | K38 | L42 | M72 | T02 | V20 | W23 | X10 |
| A04 | B22 | G05 | H03 | J37 | K40 | L45 | M73 | T03 | V22 | W24 | X14 |
| A05 | B23 | G06 | H04 | J52 | K41 | L46 | M75 | T05 | V23 | W25 | X15 |
| A06 | B25 | G07 | H05 | J54 | K42 | L48 | N05 | T07 | V24 | W27 | X16 |
| A07 | B27 | G08 | H06 | J55 | K43 | L49 | N06 | T08 | V25 | W28 | X17 |
| A08 | B28 | G09 | H07 | J56 | K44 | L50 | N26 | T09 | V26 | W30 | X19 |
| A09 | B29 | G10 | H08 | J57 | K45 | L51 | O05 | T10 | V27 | W37 | X20 |
| A10 | B38 | G11 | H09 | J58 | K46 | L52 | O06 | T14 | V28 | W38 | X21 |
| A11 | B39 | G13 | H10 | J59 | K47 | L53 | O07 | T15 | V29 | W39 | X22 |
| A12 | B41 | G21 | H11 | J60 | K48 | L56 | O08 | T16 | V30 | W40 | X23 |
| A16 | C01 | G23 | H12 | J61 | K52 | L57 | O09 | T17 | V31 | W41 | X24 |
| A17 | C05 | G24 | H13 | J63 | K53 | L58 | O10 | T28 | V32 | W42 | X25 |
| A18 | D10 | G25 | H14 | J65 | K54 | L59 | O15 | T30 | V33 | W43 | X45 |
| A20 | D19 | G27 | H15 | J68 | K55 | L60 | O17 | T32 | V34 | W44 |  |
| A22 | E01 | G28 | H16 | J69 | K56 | L62 | O18 | T33 | V35 | W45 |  |
| A24 | E12 | G29 | H17 | J70 | K57 | L65 | O21 | T34 | V36 | W46 |  |
| A25 | E13 | G30 | H19 | J72 | K66 | L67 | O22 | T36 | V37 | W47 |  |
| A26 | E19 | G31 | H29 | K01 | K67 | L68 | O23 | T37 | V38 | W48 |  |
| A27 | E21 | G32 | H30 | K02 | K69 | L69 | O24 | T38 | V39 | W49 |  |
| A28 | E23 | G33 | H32 | K04 | K71 | L70 | O25 | T39 | V40 | W50 |  |
| A29 | E28 | G34 | H33 | K05 | L01 | L74 | O26 | T41 | V41 | W51 |  |
| A30 | E29 | G35 | H34 | K06 | L02 | L75 | O27 | T50 | V42 | W52 |  |
| A31 | E30 | G36 | H35 | K07 | L03 | L77 | O29 | T51 | V43 | W53 |  |
| A32 | E31 | G38 | H36 | K08 | L04 | L79 | O32 | T52 | V44 | W54 |  |
| A34 | E33 | G40 | H37 | K09 | L05 | L80 | P05 | T53 | V45 | W55 |  |
| A36 | E39 | G41 | H40 | K10 | L06 | L81 | P17 | T56 | V46 | W56 |  |
| A38 | E40 | G48 | H41 | K11 | L07 | L90 | P18 | T76 | V48 | W57 |  |
| A39 | E41 | G49 | H46 | K12 | L08 | M01 | P20 | T77 | V49 | W58 |  |
| A40 | E42 | G50 | H47 | K13 | L09 | M02 | P31 | T85 | V51 | W60 |  |
| A41 | E43 | G51 | H49 | K14 | L10 | M03 | Q01 | T89 | V52 | W61 |  |
| A42 | E44 | G52 | H62 | K15 | L12 | M04 | Q07 | T94 | V54 | W62 |  |
| A43 | E46 | G53 | J01 | K17 | L13 | M05 | Q08 | V01 | V56 | W63 |  |
| A44 | E47 | G57 | J02 | K18 | L16 | M06 | Q22 | V02 | V58 | W64 |  |
| A45 | E52 | G58 | J03 | K19 | L18 | M08 | Q23 | V03 | V60 | W65 |  |
| A47 | E53 | G59 | J04 | K20 | L19 | M17 | Q24 | V04 | V66 | W67 |  |
| A48 | E54 | G60 | J05 | K22 | L20 | M18 | Q25 | V05 | V67 | W80 |  |
| A49 | E55 | G61 | J07 | K23 | L21 | M20 | Q43 | V06 | V68 | W93 |  |
| A51 | E57 | G63 | J08 | K24 | L22 | M21 | Q44 | V07 | W05 | W94 |  |
| A57 | E59 | G67 | J16 | K25 | L23 | M22 | Q45 | V08 | W06 | W95 |  |
| B01 | E61 | G68 | J18 | K26 | L25 | M23 | Q47 | V09 | W08 | W96 |  |
| B02 | E62 | G69 | J19 | K27 | L26 | M24 | R09 | V10 | W09 | W97 |  |
| B04 | E64 | G70 | J20 | K28 | L27 | M25 | S17 | V11 | W10 | W98 |  |
| B06 | F22 | G71 | J21 | K29 | L28 | M34 | S18 | V12 | W15 | X01 |  |
| B08 | F23 | G72 | J23 | K30 | L29 | M35 | S19 | V13 | W16 | X02 |  |
| B09 | F28 | G73 | J27 | K31 | L30 | M36 | S20 | V14 | W17 | X03 |  |
| B10 | F38 | G74 | J28 | K32 | L33 | M37 | S29 | V15 | W18 | X04 |  |
| B12 | F39 | G76 | J29 | K33 | L34 | M51 | S32 | V16 | W19 | X05 |  |
| B14 | G01 | G78 | J30 | K34 | L37 | M52 | S54 | V17 | W20 | X07 |  |

Note: The codes M24, R09, S29 and S32 had not been assigned to a surgical procedure category by Abbotts et al,. These codes were assigned to the restrictive category by the authors following review of all codes, cross reference with the Abbotts et al., categorisation approach as per their supplementary file and consensus from clinical experts in those specialities.

# **Supplementary material Table S2: OPCS-4.10 codes included in the intermediate A category (Surgery under general anaesthesia)**

All codes below were assigned a surgical carbon emission factor for surgery under general anaesthesia. This included anaesthetic gas emissions, energy use adjusted for length of procedure (determined using the Theatre Productivity Data Collection, where available), consumables, equipment, water, and waste

| A13 | C31 | E11 | F52 | M07 | M76 | O38 | Q30 | S21 | T23 | T97 | W75 |
| --- | --- | --- | --- | --- | --- | --- | --- | --- | --- | --- | --- |
| A14 | C32 | E14 | F53 | M09 | M79 | O39 | Q31 | S22 | T24 | T98 | W76 |
| A33 | C33 | E15 | G17 | M10 | M81 | O40 | Q32 | S23 | T25 | V21 | W77 |
| A59 | C34 | E16 | G26 | M19 | M83 | P01 | Q34 | S24 | T26 | V47 | W78 |
| A60 | C35 | E17 | G75 | M26 | N01 | P03 | Q35 | S25 | T27 | V69 | W79 |
| A61 | C37 | E20 | H42 | M27 | N03 | P07 | Q36 | S26 | T29 | V70 | W81 |
| A62 | C86 | E27 | H44 | M28 | N07 | P19 | Q37 | S27 | T31 | W01 | W82 |
| A63 | D01 | E38 | H48 | M29 | N08 | P21 | Q38 | S28 | T42 | W02 | W83 |
| A64 | D03 | F01 | H50 | M32 | N09 | P22 | Q39 | S30 | T43 | W03 | W84 |
| A75 | D12 | F03 | H51 | M33 | N10 | P23 | Q49 | S31 | T48 | W04 | W85 |
| B17 | D13 | F04 | H55 | M38 | N11 | P24 | Q50 | S33 | T55 | W07 | W86 |
| B30 | D14 | F09 | H56 | M39 | N13 | P25 | Q52 | S34 | T57 | W11 | W87 |
| B31 | D15 | F11 | H57 | M41 | N15 | P28 | Q54 | S35 | T64 | W12 | W88 |
| B33 | D16 | F18 | H58 | M42 | N18 | P30 | Q57 | S36 | T65 | W13 | W89 |
| B34 | D17 | F24 | H59 | M43 | N22 | P32 | R01 | S37 | T67 | W14 | X11 |
| B35 | D20 | F26 | H60 | M44 | N27 | Q05 | R02 | S38 | T68 | W26 | X12 |
| B36 | D22 | F29 | H66 | M48 | N28 | Q09 | R17 | S39 | T69 | W31 | X27 |
| B37 | D23 | F30 | J11 | M53 | N29 | Q10 | R18 | S48 | T70 | W32 | X53 |
| C02 | D24 | F32 | J62 | M54 | N30 | Q11 | R28 | S49 | T71 | W33 | X55 |
| C03 | D26 | F34 | J73 | M55 | N32 | Q16 | R29 | S56 | T72 | W59 |  |
| C06 | D28 | F36 | K16 | M56 | O01 | Q17 | R30 | S57 | T74 | W68 |  |
| C08 | E02 | F40 | K62 | M57 | O02 | Q19 | R34 | S62 | T79 | W69 |  |
| C09 | E03 | F42 | L76 | M58 | O03 | Q20 | S01 | S63 | T80 | W70 |  |
| C24 | E04 | F44 | L89 | M60 | O04 | Q26 | S02 | T19 | T83 | W71 |  |
| C25 | E07 | F45 | L93 | M64 | O19 | Q27 | S03 | T20 | T87 | W72 |  |
| C26 | E08 | F46 | L97 | M65 | O35 | Q28 | S04 | T21 | T91 | W73 |  |
| C27 | E10 | F50 | L98 | M66 | O37 | Q29 | S05 | T22 | T92 | W74 |  |

Note: The codes M07, O35, O37, O38, O39, O40, V69, V70 had not been assigned to a surgical procedure category by Abbotts et al,. These codes were assigned to the intermediate A category by the authors following review of all codes, cross reference with the Abbotts et al., categorisation approach as per their supplementary file and consensus from clinical experts in those specialities.

# **Supplementary material Table S3: OPCS-4.10 codes included in the intermediate B category (Surgery under local or regional anaesthesia)**

All codes below were assigned a surgical carbon emission factor for surgery under local or regional anaesthesia. This excluded anaesthetic gas emissions and included energy use adjusted for length of procedure (determined using the Theatre Productivity Data Collection, where available), consumables, equipment, water, and waste.

| A65 | C13 | C47 | C72 | D08 | G12 | J77 | L54 | M16 | P15 | S41 | T88 |
| --- | --- | --- | --- | --- | --- | --- | --- | --- | --- | --- | --- |
| A66 | C14 | C49 | C73 | E05 | G14 | K35 | L63 | M49 | P29 | S42 | T96 |
| A67 | C15 | C51 | C74 | E09 | G20 | K59 | L66 | M67 | Q02 | S47 | V62 |
| A68 | C16 | C52 | C75 | E24 | H52 | K60 | L71 | M68 | Q41 | S60 | W29 |
| A69 | C17 | C53 | C77 | E34 | H53 | K64 | L73 | M70 | Q51 | S64 | W34 |
| A70 | C18 | C54 | C79 | E35 | H54 | K65 | L82 | M71 | Q56 | S66 | W91 |
| A73 | C19 | C55 | C80 | E48 | J06 | K68 | L83 | M86 | R04 | S68 | W92 |
| A76 | C20 | C57 | C81 | E50 | J10 | K72 | L84 | N17 | R05 | S70 | W99 |
| A77 | C22 | C59 | C82 | E63 | J12 | K73 | L85 | N19 | R06 | T11 | X46 |
| A78 | C23 | C60 | C83 | E65 | J13 | K74 | L86 | N20 | R07 | T12 |  |
| A79 | C29 | C61 | C84 | E66 | J15 | K75 | L87 | N24 | R08 | T13 |  |
| A81 | C39 | C62 | C85 | E67 | J24 | K76 | L88 | N34 | R10 | T45 |  |
| A82 | C40 | C64 | C88 | F02 | J25 | K77 | L91 | O20 | R11 | T54 |  |
| A84 | C41 | C65 | C89 | F05 | J26 | L31 | L94 | P06 | R12 | T59 |  |
| B40 | C43 | C66 | C91 | F06 | J34 | L35 | L96 | P09 | S06 | T60 |  |
| C10 | C44 | C67 | D02 | F48 | J35 | L39 | L99 | P11 | S10 | T61 |  |
| C11 | C45 | C69 | D04 | F51 | J36 | L43 | M13 | P13 | S11 | T62 |  |
| C12 | C46 | C71 | D06 | F58 | J49 | L47 | M15 | P14 | S40 | T86 |  |

Note: The codes C91, R11 and O20 had not been assigned to a surgical procedure category by Abbotts et al,. These codes were therefore assigned to the intermediate B category by the authors following review of all codes, cross reference with the Abbotts et al., categorisation approach as per their supplementary file and consensus from clinical experts in those specialities.

# **Supplementary material Table S4: OPCS-4.10 codes included in the inclusive A and B categories (Procedure performed outside of theatre in a specialist suite, unit, procedure room, ward or other clinic setting.)**

All codes below were assigned a carbon emission factor based on available published literature (endoscopy carbon factor) or calculated using the length of procedure data were available, adjusted energy use based on clinical knowledge of the setting and anaesthetic requirements for the procedure.

| A52 | E37 | F55 | G47 | H23 | J14 | J48 | K61 | P27 | R21 | S44 | W90 |
| --- | --- | --- | --- | --- | --- | --- | --- | --- | --- | --- | --- |
| A53 | E49 | F56 | G54 | H24 | J17 | J50 | K78 | Q03 | R22 | S45 | X41 |
| A54 | E51 | F63 | G55 | H25 | J38 | J51 | L72 | Q12 | R25 | S50 | X42 |
| A55 | F08 | G15 | G62 | H26 | J39 | J53 | L95 | Q13 | R27 | S51 | X59 |
| B32 | F10 | G16 | G64 | H27 | J40 | J66 | M11 | Q14 | R32 | S52 |  |
| C04 | F12 | G18 | G65 | H28 | J41 | J67 | M12 | Q15 | S07 | S53 |  |
| C48 | F13 | G19 | G79 | H31 | J42 | J74 | M14 | Q18 | S08 | T46 |  |
| D05 | F14 | G42 | G80 | H68 | J43 | J76 | M30 | Q21 | S09 | T81 |  |
| D07 | F15 | G43 | H18 | H69 | J44 | K49 | M31 | Q48 | S13 | V50 |  |
| E06 | F16 | G44 | H20 | H70 | J45 | K50 | M45 | Q55 | S14 | W35 |  |
| E25 | F17 | G45 | H21 | H71 | J46 | K51 | M77 | R13 | S15 | W36 |  |
| E36 | F20 | G46 | H22 | J09 | J47 | K58 | M85 | R14 | S43 | W66 |  |

Note: The codes H71 and R13 had not been assigned to a surgical procedure category by Abbotts et al,. These codes were assigned to the inclusive A and B categories by the authors following review of all codes, cross reference with the Abbotts et al., categorisation approach as per their supplementary file and consensus from clinical experts in those specialities.

# **Supplementary material Table S5: OPCS-4.10 codes included in the Non-surgical intervention or treatment, outside of theatre A and B categories (Procedure, treatment, or intervention performed on a specialist unit and or requiring specialist input and procedure, treatment, or intervention that can be delivered on the ward and is deemed part of ward-based care)**

| The following codes where assigned a carbon emission factor based on available published literature (childbirth (delivery), radiotherapy, dialysis, blood transfusion) or calculated using the length of procedure data were available and adjusted based on clinical knowledge of the setting and anaesthetic requirements for the procedure. | | | | | | | | | | | |
| --- | --- | --- | --- | --- | --- | --- | --- | --- | --- | --- | --- |
| A83 | Q58 | R19 | R20 | R23 | R24 | X32 | X33 | X40 | X47 | X65 | X69 |
| The following codes were excluded from carbon factor allocation as there was no available appropriate carbon emission factor available to assign to these. Most represent pharmacological treatment (all but S12) which is not captured in the inpatient bed day carbon emission factor. | | | | | | | | | | | |
| C90 | X29 | X35 | X39 | X71 | X74 | X84 | X86 | X89 | X91 | X93 | X96 |
| S12 | X30 | X37 | X44 | X72 | X82 | X85 | X87 | X90 | X92 | X95 | X98 |
| X28 | X34 | X38 | X70 | X73 | X83 |  |  |  |  |  |  |
| The following codes did not have a specific carbon factor applied to them as these represent care that would form part of ward (level 1, 2 and 3) based care and therefore captured / included in the carbon emissions allocated to the inpatient bed day. | | | | | | | | | | | |
| E85 | E91 | F43 | N35 | S58 | U50 | U53 | X48 | X51 | X58 | X62 | X67 |
| E87 | E97 | L92 | P26 | S59 | U51 | U54 | X49 | X52 | X60 | X66 | X68 |
| E89 | E98 | M47 | R15 | U34 | U52 | X43 | X50 | X56 | X61 |  |  |

# **Supplementary material Table S6: OPCS-4.10 codes included in diagnostic test category.**

| All codes below were assigned a carbon emission factor based on available published literature (imaging, blood test). | | | | | | | | | | | |
| --- | --- | --- | --- | --- | --- | --- | --- | --- | --- | --- | --- |
| C87 | R37 | R43 | U04 | U07 | U10 | U13 | U16 | U20 | U29 | U35 | U37 |
| K63 | R38 | T90 | U05 | U08 | U11 | U14 | U17 | U21 | U32 | U36 | X31 |
| R36 | R42 | U01 | U06 | U09 | U12 | U15 | U18 | U23 |  |  |  |
| The following codes were excluded due to a lack of published available carbon factors. | | | | | | | | | | | |
| E92 | E94 | R40 | U22 | U25 | U27 | U30 | U31 | U33 | U38 | U40 | U41 |
| E93 | E95 | U19 | U24 | U26 | U28 |  |  |  |  |  |  |

# **Supplementary material Table S7: OPCS-4.10 codes excluded as deemed subsidiary / qualifying.**

| **Definition** | **Description** | **Codes** |
| --- | --- | --- |
| Subsidiary Classification of Methods of Operation’ | These are used as supplementary or qualifying codes to provide additional information about the approach, technique, or device used in a procedure. | Y01-99, O44, O48 |
| Subsidiary Classification of Sites of Operation’ | These are used as supplementary or qualifying codes to indicate various factors related to the procedure, such as patient status, location, or specific circumstances. | Z01-99, O11-O14, O16, O28, O30-O31, O33-O34, O36, O42-O43, O45-O47, O50, O52-O53, V55 |

# **Supplementary material Table S8: Carbon factors applied to each activity element and information source**

| Activity element | Carbon factor (KgCO_2_e) | Mapped to activity | Carbon factor inclusions and exclusions | Source |
| --- | --- | --- | --- | --- |
| Low intensity bed-day | 37.9 | Applied to general and acute ward stay activity | Includes: Ward-based consumables; Ward-based electricity, fuel and water; Shared services electricity, fuel and water; Patient food consumption; Linen use and cleaning; Facilities cleaning; Staff and visitor travel; Waste; Sterilisation; Administration activities.  Excludes: Pharmaceuticals; Capital goods (e.g. buildings, car parks); Staff training; Health authorities and financial services; Patient travel; Surgical procedure. | Sustainable Healthcare Coalition |
| High intensity bed-day | 89.5 | Applied to critical care stay activity | Includes: Ward-based consumables; Ward-based electricity, fuel and water; Shared services electricity, fuel and water; Patient food consumption; Linen use and cleaning; Facilities cleaning; Staff and visitor travel; Waste; Sterilisation; Administration activities.  Excludes: Pharmaceuticals; Capital goods (e.g. buildings, car parks); Staff training; Health authorities and financial services; Patient travel; Surgical procedure. | Sustainable Healthcare Coalition |
| Face to face out-patient attendances | 22.0 | Applied to all face-to-face attendances | Includes: NHS carbon footprint and carbon footprint plus categories.  A return patient journey is included.  Expenditure data is used to apportion emissions to hospital activity. | University of Kent |
| Virtual out-patient attendances | 0.1 | Applied to all virtual attendances | Includes: Scope 2 and scope 3 emissions (life cycle assessment) of a phone call. | Greener NHS |
| Patient travel per mile | 0.25 | Applied to all in-patient elective spells where specialty-level travel distance is available | Includes: Scope 1 - Emissions associated with the combustion of fuel (petrol, diesel, etc).  Scope 3 - Well-to-tank emissions associated with the volume of fuel consumed (transportation, refining, etc).  Excludes: Manufacturing (embodied carbon) | Greener NHS and data from the Hospital Episodes Statistics dataset |
| Round-trip for in-patient stay | 4.4 | Applied to all in-patient elective spells where specialty-level travel distance is not available | Includes: Scope 1 - Emissions associated with the combustion of fuel (petrol, diesel, etc).  Scope 3 - Well-to-tank emissions associated with the volume of fuel consumed (transportation, refining, etc).  Excludes: Manufacturing (embodied carbon) | Greener NHS |
| Emergency department visit | 13.8 | Applied to all non-elective inpatient spells | Includes: Consumables and equipment used during triage and treatment, Facilities data (fuels, electricity, water use and waste generated) for the ED, Staff travel, Shared hospital services, Cleaning and Sterilisation  Excludes: Pharmaceuticals administered during patient treatment, Capital goods (eg buildings, car parks), staff training, Health authorities and financial services, Patient travel | Sustainable Healthcare Coalition |
| Round trip for emergency department visit | 2.25 | Applied to all non-elective inpatient spells | Includes: Scope 1 - Emissions associated with the combustion of fuel (petrol, diesel, etc).  Scope 3 - Well-to-tank emissions associated with the volume of fuel consumed (transportation, refining, etc).  Excludes: Manufacturing (embodied carbon) | Greener NHS |
| Average surgical procedure  without desflurane use  (60 minutes) | 38.4    Electricity footprint is adjusted by length of procedure (where data is available) | Surgical procedure, Intermediate A and Restrictive category | Includes: Anaesthetic Room & Operating Room consumables, equipment, electricity, fuel and water; Shared Services electricity, fuel and water; Anaesthetic gases (except desflurane) and direct emissions; Staff travel; Waste; Cleaning and Sterilisation.  Excludes: Desflurane given very limited use within the NHS during the study period; Pharmaceuticals; Implants & prosthetics; Blood or other surgery specific items; Capital goods (eg buildings, car parks); Surgeon training; Other staff training; Health authorities and financial services; Patient travel. | Sustainable Healthcare Coalition |
| Average surgical procedure - without anesthetic gas use  (60 minutes) | 21.4    Electricity footprint is adjusted by length of procedure (where data is available) | Surgical procedure, Intermediate B and Inclusive A category (except Endoscopy) | Includes: Anaesthetic Room & Operating Room consumables, equipment, electricity, fuel and water; Shared Services electricity, fuel and water; Staff travel; Waste; Cleaning and Sterilisation.  Excludes: Anaesthetic gases and direct emissions; Pharmaceuticals; Implants & prosthetics; Blood or other surgery specific items; Capital goods (eg buildings, car parks); Surgeon training; Other staff training; Health authorities and financial services; Patient travel. | Sustainable Healthcare Coalition |
| Average surgical procedure – without anesthetic gas use and energy reduction to reflect out of theatre setting  (60 minutes) | 11.6    Theatre electricity footprint is reduced by x6. Electricity footprint is adjusted by length of procedure (where data is available) | Surgical procedure, Inclusive B category | Includes: Anaesthetic Room & Operating Room consumables, equipment, electricity, fuel and water; Shared Services electricity, fuel and water; Staff travel; Waste; Cleaning and Sterilisation.  Excludes: Anaesthetic gases and direct emissions; Pharmaceuticals; Implants & prosthetics; Blood or other surgery specific items; Capital goods (eg buildings, car parks); Surgeon training; Other staff training; Health authorities and financial services; Patient travel. | Sustainable Healthcare Coalition and MacNeill et al, 2017^1^ |
| Average surgical procedure –  without consumables, equipment, water and waste  (60 minutes) | 31.1    Electricity footprint is adjusted by length of procedure (where data is available) | Non-surgical intervention or treatment, outside of theatre category A - Electroconvulsive therapy | Includes: Anaesthetic Room & Operating Room electricity and fuel; Shared Services electricity and fuel; Anaesthetic gases (except desflurane) and direct emissions; Staff travel.  Excludes: Desflurane given very limited use within the NHS during the study period; Anaesthetic Room & Operating Room consumables, equipment, water and waste; Pharmaceuticals; Implants & prosthetics; Blood or other surgery specific items; Capital goods (eg buildings, car parks); Surgeon training; Other staff training; Health authorities and financial services; Patient travel. | Sustainable Healthcare Coalition |
| Endoscopy | 15.4 | Surgical procedure, Inclusive A category – Endoscopy | Includes: Energy; consumables; medical and non-medical equipment; medical gases; waste; freight;  Excludes; Travel (patient and staff) | Lacroute et al, 2023^2^ |
| Radiotherapy | 30.6 | Non-surgical intervention or treatment, outside of theatre A - Radiotherapy | Inclusion: Patient travel; Pre-treatment imaging; Linac energy electricity - treatment; Linac power electricity – idle; SF6 leakage; PPE | Chuter et al, 2023^3^ |
| Dialysis | 14.8 | Non-surgical intervention or treatment, outside of theatre A – Dialysis | Includes: Utilities (electricity and heat from combined heat and power (CHP) plant, water supply); Pharmaceuticals; Consumables and Equipment; Waste treatment (solid waste, wastewater treatment)  Excludes; Travel (patient and staff) | Sustainable Healthcare Coalition^4^ |
| Childbirth (delivery) | 17.0 | Non-surgical intervention or treatment, outside of theatre A – Childbirth (delivery) | Includes: Energy (electricity and natural gas) – extraction and processing, generation, use (HVAC, lighting, machines), consumables and instruments (raw material extraction, manufacture, sterilisation, disposal of waste (landfill, incineration, autoclave and landfill)  Excludes: Manufacturing of building materials, electricity of hot water, pharmaceuticals, cleaning products (chemical manufacturing) | Campion et al, 2012^6^ |
| Blood transfusion | 7.56 | Non-surgical intervention or treatment, outside of theatre B – Blood transfusion | Includes: Life cycle analysis - Donation (machines and consumables); Transportation (donors, blood, consumables delivery); Manufacturing (machines, consumables); Testing (machines, consumables), Stockholding units (machines); Transfusion (machines, consumables, refrigerant leak); Disposal (incineration). | Hibbs et al, 2024^5^ |
| Blood test | 0.3 | Diagnostic tests | Includes: Average of life cycle assessment of four key pathology tests. Consumables and waste for venepuncture and laboratory analyses, and electricity and water use for laboratory analyses.  Excludes: arterial blood gas assessment | McAlister et al, 2020^7^ |
| Computerised tomography (CT) scan | 9.2  CT scan carbon factor applied across all radiological imaging as CT imaging was the most frequent procedure code for radiological imaging. | Radiological imaging and investigations | Includes: Scanner electricity use and all consumables and associated waste, including bedding, imaging contrast, and gloves. | McAlister et al, 2022^8^ |

**References**

1. MacNeill AJ, Lillywhite R, Brown CJ. The impact of surgery on global climate: a carbon footprinting study of operating theatres in three health systems. *Lancet Planet Health* 2017; **1**(9): e381-e8.

2. Lacroute J, Marcantoni J, Petitot S, et al. The carbon footprint of ambulatory gastrointestinal endoscopy. *Endoscopy* 2023; **55**(10): 918-26.

3. Chuter R, Stanford-Edwards C, Cummings J, et al. Towards estimating the carbon footprint of external beam radiotherapy. *Phys Med* 2023; **112**: 102652.

4. Sustainable Healthcare Coalition. A care pathway environmental assessment of haemodialysis at The Newcastle upon Tyne Hospitals NHS Foundation trust. 2022. <https://shcoalition.org/wp-content/uploads/2022/06/ICHD_CarePathway_-220610.pdf> (accessed 22nd April 2024).

5. Hibbs SP, Thomas S, Agarwal N, et al. What is the environmental impact of a blood transfusion? A life cycle assessment of transfusion services across England. *Transfusion* 2024; **64**(4): 638-45.

6. Campion N, Thiel CL, DeBlois J, Woods NC, Landis AE, Bilec MM. Life cycle assessment perspectives on delivering an infant in the US. *Sci Total Environ* 2012; **425**: 191-8.

7. McAlister S, Barratt AL, Bell KJ, McGain F. The carbon footprint of pathology testing. *Med J Aust* 2020; **212**(8): 377-82.

8. McAlister S, McGain F, Petersen M, et al. The carbon footprint of hospital diagnostic imaging in Australia. *Lancet Reg Health West Pac* 2022; **24**: 100459.

# **Supplementary material Table S9: Carbon footprint (kilotonnes CO_2_e) breakdown by specialty and activity type**

| **Specialty** | **Outpatient attendance** | **Bed days** | **Emergency department visit** | **Patient travel (admitted patient care only)** | **Surgical procedure, non-surgical intervention, or treatment** | **Diagnostic test** | **Total** | **Per patient (kg CO_2_e)** |
| --- | --- | --- | --- | --- | --- | --- | --- | --- |
| General Internal Medicine | 58.252 | 450.155 | 24.577 | 18.041 | 6.183 | 4.941 | 562.150 | 92.476 |
| Trauma & Orthopaedics | 135.563 | 127.932 | 3.986 | 5.197 | 17.779 | 0.884 | 291.341 | 36.754 |
| General Surgery | 98.06 | 139.939 | 9.682 | 10.305 | 26.908 | 2.443 | 287.336 | 38.365 |
| Paediatrics | 67.295 | 126.319 | 16.01 | 11.332 | 1.955 | 0.535 | 223.445 | 45.159 |
| Allied Health Professional | 198.748 | 1.879 | 0.031 | 0.051 | 0.171 | 0.005 | 200.886 | 18.874 |
| Geriatric Medicine | 8.176 | 163.253 | 5.501 | 3.757 | 0.404 | 1.506 | 182.597 | 196.006 |
| Ophthalmology | 136.049 | 1.862 | 0.224 | 2.643 | 9.179 | 0.041 | 149.998 | 20.904 |
| Obstetrics | 60.321 | 43.562 | 9.504 | 6.576 | 10.148 | 0.283 | 130.394 | 34.194 |
| Gynaecology | 66.87 | 30.772 | 6.094 | 4.948 | 11.727 | 0.205 | 120.616 | 28.002 |
| Cardiology | 57.022 | 45.065 | 2.174 | 2.915 | 2.295 | 1.791 | 111.262 | 27.934 |
| Midwifery | 87.569 | 13.12 | 3.585 | 2.408 | 2.185 | 0.025 | 108.892 | 24.474 |
| Adult Mental Illness | 22.904 | 81.088 | 0.357 | 0.295 | 0.006 | 0.001 | 104.65 | 79.225 |
| Nursing | 99.371 | 4.233 | 0.052 | 0.22 | 0.377 | 0.014 | 104.267 | 15.991 |
| Respiratory Medicine | 34.364 | 62.274 | 2.626 | 2.337 | 1.082 | 0.572 | 103.254 | 39.700 |
| Urology | 42.97 | 23.918 | 2.017 | 3.595 | 9.65 | 0.403 | 82.553 | 22.710 |
| Gastroenterology | 28.396 | 31.264 | 1.162 | 5.046 | 14.426 | 0.28 | 80.573 | 21.244 |
| Acute Internal Medicine | 3.469 | 65.485 | 4.503 | 3.099 | 0.26 | 0.761 | 77.578 | 142.286 |
| Ear Nose and Throat | 51.148 | 11.325 | 1.21 | 1.748 | 6.247 | 0.093 | 71.771 | 24.041 |
| Clinical Haematology | 41.482 | 19.722 | 0.484 | 3.558 | 1.572 | 0.175 | 66.992 | 17.946 |
| Renal Medicine | 21.964 | 24.184 | 0.973 | 3.758 | 12.44 | 0.176 | 63.495 | 29.519 |
| Dermatology | 60.302 | 0.497 | 0.009 | 0.706 | 1.755 | 0.004 | 63.272 | 19.743 |
| Endocrinology and Diabetes | 18.331 | 37.748 | 1.642 | 1.296 | 0.187 | 0.512 | 59.717 | 39.781 |
| Neurology | 31.397 | 18.962 | 0.477 | 1.424 | 0.387 | 0.33 | 52.976 | 23.647 |
| Rheumatology | 38.768 | 7.689 | 0.37 | 0.925 | 0.139 | 0.089 | 47.98 | 18.194 |
| Clinical Oncology | 33.367 | 8.216 | 0.533 | 3.128 | 2.642 | 0.129 | 48.016 | 16.435 |
| Medical Oncology | 27.626 | 9.686 | 0.538 | 2.945 | 0.577 | 0.181 | 41.553 | 15.793 |
| Emergency Medicine | 0 | 30.28 | 6.106 | 4.098 | 0.36 | 0.803 | 41.648 | 93.483 |
| Radiology | 34.695 | 1.054 | 0.028 | 0.365 | 1.066 | 0.07 | 37.278 | 22.186 |
| Forensic Psychiatry | 0.38 | 36.661 | 0.007 | 0.013 | 0 | 0 | 37.061 | 1838.778 |
| Plastic Surgery | 22.846 | 6.056 | 0.687 | 1.63 | 5.064 | 0.013 | 36.296 | 26.321 |
| Neurosurgery | 7.57 | 24.446 | 0.416 | 0.79 | 1.744 | 0.24 | 35.206 | 58.283 |
| Anaesthetics | 22.659 | 6.527 | 0.1 | 0.736 | 1.624 | 0.046 | 31.693 | 19.242 |
| Not recorded | 18.537 | 10.307 | 0.127 | 0.108 | 0.062 | 0.005 | 29.146 | 31.386 |
| Cardiothoracic Surgery | 3.532 | 21.347 | 0.188 | 0.596 | 1.781 | 0.083 | 27.528 | 99.828 |
| Oral Surgery | 17.948 | 1.698 | 0.14 | 0.639 | 2.616 | 0.01 | 23.051 | 23.158 |
| Rehabilitation Medicine | 0 | 22.032 | 0.221 | 0.202 | 0.032 | 0.036 | 22.523 | 961.912 |
| Old Age Psychiatry | 3.45 | 18.299 | 0.045 | 0.04 | 0.004 | 0 | 21.838 | 104.905 |
| Oral and Maxillofacial Surgery | 14.171 | 2.662 | 0.267 | 0.511 | 2.313 | 0.019 | 19.944 | 24.908 |
| Intensive Care Medicine | 0.235 | 13.032 | 0.266 | 0.182 | 0.195 | 0.088 | 13.998 | 368.254 |
| Child and Adolescent Psychiatry | 8.324 | 3.318 | 0.007 | 0.009 | 0 | 0 | 11.658 | 22.431 |
| Infectious Diseases | 4.25 | 5.07 | 0.241 | 0.202 | 0.042 | 0.056 | 9.861 | 36.682 |
| Audio Vestibular Medicine | 9.695 | 0.003 | 0 | 0.001 | 0.003 | 0.001 | 9.703 | 20.288 |
| Paediatric Surgery | 4.043 | 3.694 | 0.226 | 0.446 | 1.144 | 0.027 | 9.579 | 34.837 |
| General Medical Practice | 2.388 | 6.845 | 0.122 | 0.093 | 0.027 | 0.004 | 9.478 | 78.129 |
| Orthodontics | 8.903 | 0.017 | 0.002 | 0.007 | 0.026 | 0 | 8.955 | 21.693 |
| Community Medicine | 7.1 | 0.69 | 0.012 | 0.011 | 0.01 | 0 | 7.824 | 22.461 |
| Learning Disability | 2.868 | 4.35 | 0.005 | 0.01 | 0 | 0 | 7.233 | 49.627 |
| Haematology | 4.368 | 1.456 | 0.065 | 0.676 | 0.196 | 0.017 | 6.778 | 16.783 |
| Rehabilitation | 6.677 | 0.085 | 0 | 0 | 0 | 0 | 6.763 | 19.574 |
| Restorative dentistry | 5.946 | 0.02 | 0 | 0.035 | 0.084 | 0.001 | 6.087 | 21.405 |
| Paediatric Cardiology | 3.297 | 2.099 | 0.028 | 0.128 | 0.095 | 0.066 | 5.713 | 31.865 |
| Clinical Physiology | 4.727 | 0.006 | 0 | 0.001 | 0 | 0.001 | 4.735 | 21.849 |
| Palliative Medicine | 1.593 | 2.852 | 0.043 | 0.037 | 0.006 | 0.005 | 4.536 | 32.150 |
| Genitourinary Medicine | 3.809 | 0.22 | 0.007 | 0.006 | 0.011 | 0.002 | 4.056 | 19.833 |
| Accident & emergency | 3.996 | 0.052 | 0 | 0 | 0 | 0 | 4.049 | 21.482 |
| Paediatric Dentistry | 2.288 | 0.033 | 0.001 | 0.103 | 0.257 | 0.001 | 2.683 | 20.358 |
| Dental Medicine | 2.473 | 0.01 | 0 | 0.018 | 0.048 | 0 | 2.549 | 19.427 |
| Paediatric Neurology | 1.31 | 0.982 | 0.03 | 0.148 | 0.036 | 0.027 | 2.532 | 28.613 |
| Clinical Immunology | 1.936 | 0.246 | 0.015 | 0.171 | 0.008 | 0.002 | 2.378 | 14.253 |
| Clinical Neurophysiology | 2.188 | 0.109 | 0 | 0.023 | 0.014 | 0.001 | 2.336 | 21.449 |
| Medical Ophthalmology | 1.683 | 0.066 | 0.004 | 0.024 | 0.114 | 0.001 | 1.893 | 21.953 |
| Vascular Surgery | 0.581 | 1.022 | 0.026 | 0.036 | 0.109 | 0.006 | 1.782 | 52.461 |
| Clinical Pharmacology | 0.321 | 1.231 | 0.06 | 0.05 | 0.008 | 0.012 | 1.682 | 62.882 |
| Clinical Genetics | 1.531 | 0.052 | 0.001 | 0.005 | 0.006 | 0.001 | 1.597 | 14.648 |
| Chemical Pathology | 0.982 | 0.026 | 0 | 0.014 | 0 | 0.002 | 1.024 | 9.224 |
| Medical Virology | 0.683 | 0 | 0 | 0.004 | 0 | 0 | 0.688 | 20.932 |
| Medical Psychotherapy | 0.544 | 0.005 | 0 | 0.004 | 0.002 | 0 | 0.555 | 14.060 |
| Occupational Medicine | 0.506 | 0 | 0 | 0 | 0 | 0 | 0.506 | 20.873 |
| Nuclear Medicine | 0.328 | 0.023 | 0 | 0.026 | 0.017 | 0 | 0.396 | 16.692 |
| Intellectual Disability | 0 | 0.366 | 0 | 0 | 0 | 0 | 0.366 | 52264.634 |
| Sports and Exercise Medicine | 0.34 | 0 | 0 | 0 | 0 | 0 | 0.341 | 19.538 |
| Medical Microbiology and Virology | 0.205 | 0.07 | 0.001 | 0.012 | 0.002 | 0 | 0.29 | 18.939 |
| Allergy | 0.245 | 0.001 | 0 | 0.027 | 0 | 0 | 0.274 | 16.533 |
| Special Care Dentistry | 0.238 | 0.001 | 0 | 0.01 | 0.022 | 0 | 0.271 | 22.083 |
| Periodontics | 0.218 | 0 | 0 | 0.003 | 0.013 | 0 | 0.234 | 20.694 |
| Public Health Medicine | 0.034 | 0.156 | 0.001 | 0.003 | 0.005 | 0 | 0.199 | 90.911 |
| General Pathology | 0.129 | 0.019 | 0 | 0.001 | 0.002 | 0 | 0.152 | 16.838 |
| Immunopathology | 0.114 | 0.005 | 0 | 0.02 | 0 | 0 | 0.139 | 10.170 |
| Community Health Services Dental | 0.07 | 0.001 | 0 | 0.009 | 0.023 | 0 | 0.103 | 21.452 |
| Tropical Medicine | 0.013 | 0.041 | 0.002 | 0.001 | 0.001 | 0 | 0.059 | 76.332 |
| Medical Microbiology | 0.039 | 0.001 | 0 | 0 | 0 | 0 | 0.041 | 18.394 |
| Histopathology | 0.011 | 0.011 | 0 | 0.001 | 0.002 | 0 | 0.026 | 21.763 |
| Surgical Dentistry | 0 | 0 | 0 | 0.006 | 0.013 | 0 | 0.02 | 14.327 |
| General Dental Practice | 0.009 | 0 | 0 | 0 | 0.001 | 0 | 0.01 | 22.726 |
| Community Sexual and Reproductive Health | 0.006 | 0 | 0 | 0 | 0 | 0 | 0.006 | 5.027 |
| Blood Transfusion | 0 | 0 | 0 | 0 | 0 | 0 | 0.001 | 17.712 |

# **Supplementary material Table S10: Sensitivity analyses for specialty level totals**

|  | **Bed days carbon emissions factor +20%** | | **Bed days carbon emissions factor -20%** | | **Outpatients carbon emissions factor +20%** | | **Outpatients carbon emissions factor -20%** | |
| --- | --- | --- | --- | --- | --- | --- | --- | --- |
| **Rank** | **Clinical specialty** | **Kilotonnes CO_2_e** | **Clinical specialty** | **Kilotonnes CO_2_e** | **Clinical specialty** | **Kilotonnes CO_2_e** | **Clinical specialty** | **Kilotonnes CO_2_e** |
| 1 | General Internal Medicine | 652 | General Internal Medicine | 472 | General Internal Medicine | 574 | General Internal Medicine | 550 |
| 2 | Trauma & Orthopaedics | 317 | Trauma & Orthopaedics | 266 | Trauma & Orthopaedics | 318 | General surgery | 268 |
| 3 | General surgery | 315 | General surgery | 259 | General surgery | 307 | Trauma & Orthopaedics | 264 |
| 4 | Paediatrics | 249 | Allied Health Professional | 201 | Allied Health Professional | 241 | Paediatrics | 210 |
| 5 | Geriatric Medicine | 215 | Paediatrics | 198 | Paediatrics | 237 | Geriatric Medicine | 181 |
| 6 | Allied Health Professional | 201 | Geriatric Medicine | 150 | Geriatric Medicine | 184 | Allied Health Professional | 161 |
| 7 | Ophthalmology | 150 | Ophthalmology | 150 | Ophthalmology | 177 | Ophthalmology | 123 |
| 8 | Obstetrics | 139 | Obstetrics | 122 | Obstetrics | 142 | Obstetrics | 118 |
| 9 | Gynaecology | 127 | Gynaecology | 114 | Gynaecology | 134 | Gynaecology | 107 |
| 10 | Adult Mental Illness | 121 | Midwifery | 106 | Midwifery | 126 | Adult Mental Illness | 100 |
| 11 | Cardiology | 120 | Nursing | 103 | Nursing | 124 | Cardiology | 100 |
| 12 | Respiratory Medicine | 116 | Cardiology | 102 | Cardiology | 123 | Respiratory Medicine | 96 |
| 13 | Midwifery | 112 | Respiratory Medicine | 91 | Respiratory Medicine | 110 | Midwifery | 91 |
| 14 | Nursing | 105 | Adult Mental Illness | 88 | Adult Mental Illness | 109 | Nursing | 84 |
| 15 | Acute Internal Medicine | 91 | Urology | 78 | Urology | 91 | Acute Internal Medicine | 77 |
| 16 | Urology | 87 | Gastroenterology | 74 | Gastroenterology | 86 | Gastroenterology | 75 |
| 17 | Gastroenterology | 87 | Ear Nose and Throat | 70 | Ear Nose and Throat | 82 | Urology | 74 |
| 18 | Ear Nose and Throat | 74 | Acute Internal Medicine | 64 | Acute Internal Medicine | 78 | Ear Nose and Throat | 62 |
| 19 | Clinical Haematology | 71 | Dermatology | 63 | Dermatology | 75 | Renal Medicine | 59 |
| 20 | Renal Medicine | 68 | Clinical Haematology | 63 | Clinical Haematology | 75 | Clinical Haematology | 59 |

# **Supplementary material Figure S1: Admitted patient care volume by top 20 specialties, 2022/23.**


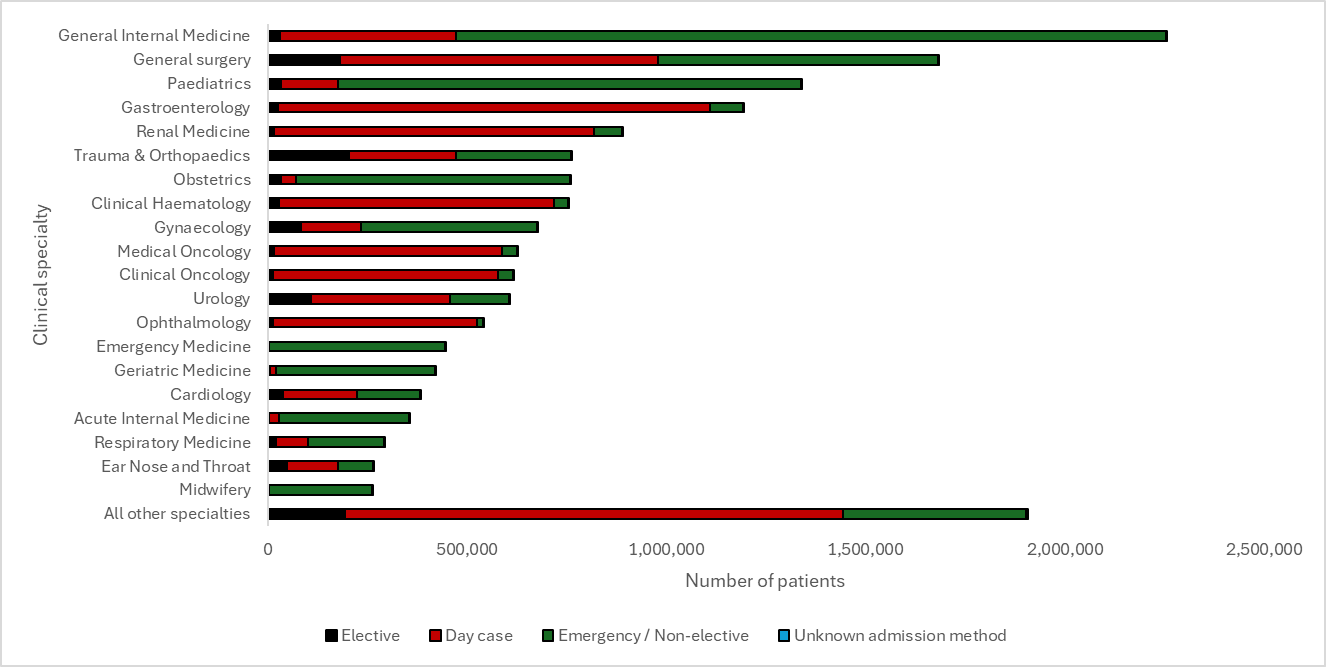


# **Supplementary material Figure S2: Outpatient attendances volume by top 20 specialties, 2022/23.**


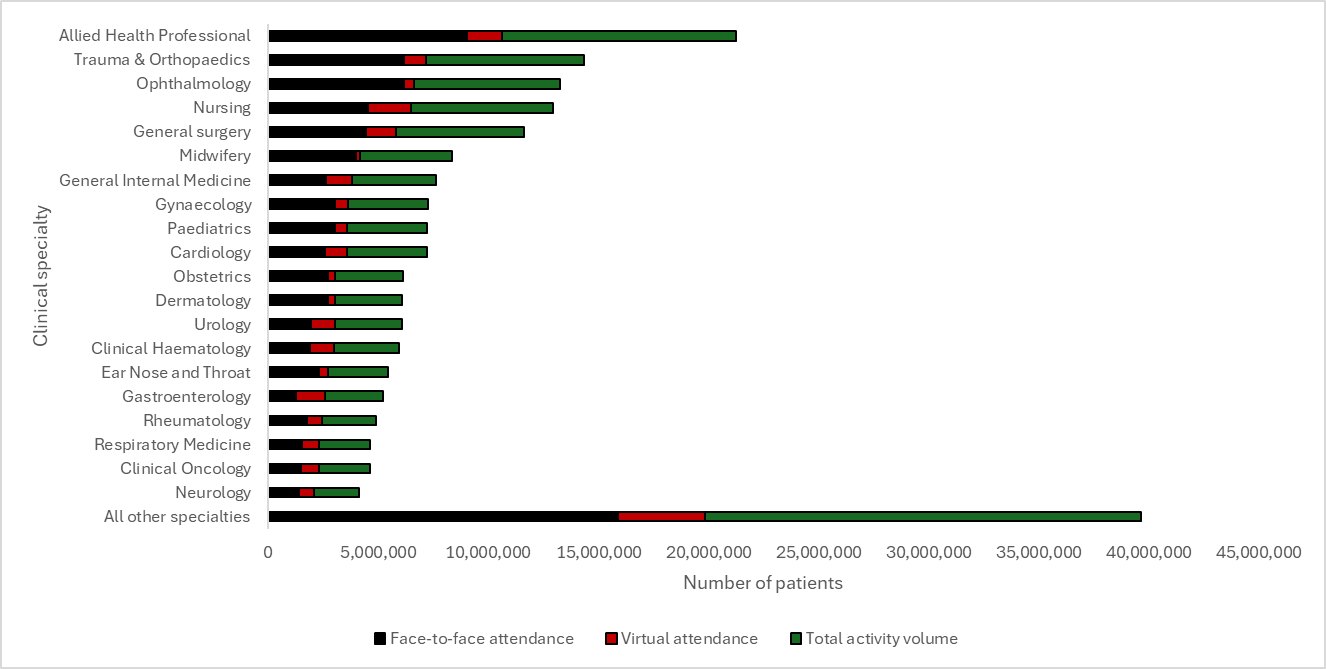


# **Supplementary material Figure S3: Carbon emissions of secondary and tertiary care in England by category.**


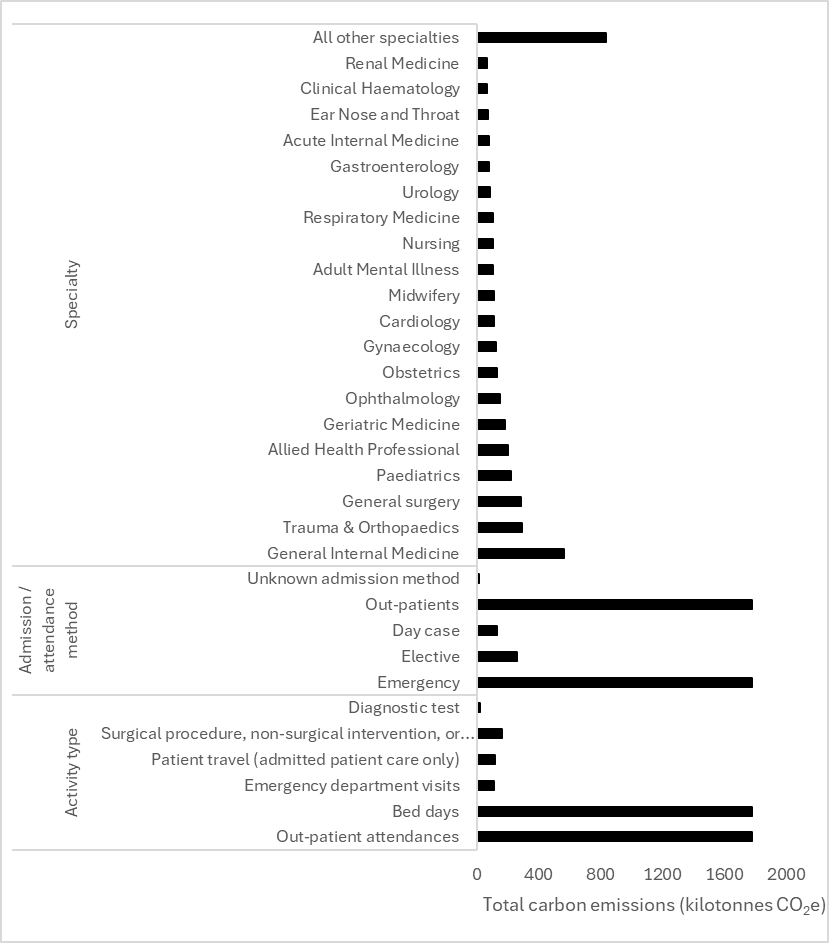

Supplement: Supplementary Figures and Tables [file mmc1.docx]
